# Evolution of the neuraminidase gene of seasonal influenza A and B viruses in Thailand between 2010 and 2015

**DOI:** 10.1371/journal.pone.0175655

**Published:** 2017-04-14

**Authors:** Nipaporn Tewawong, Preeyaporn Vichiwattana, Sumeth Korkong, Sirapa Klinfueng, Nungruthai Suntronwong, Thanunrat Thongmee, Apiradee Theamboonlers, Sompong Vongpunsawad, Yong Poovorawan

**Affiliations:** Center of Excellence in Clinical Virology, Department of Pediatrics, Faculty of Medicine, Chulalongkorn University, Bangkok, Thailand; Monash University, Australia, AUSTRALIA

## Abstract

The neuraminidase inhibitors (NAIs) oseltamivir and zanamivir are commonly used for the treatment and control of influenza A and B virus infection. However, the emergence of new influenza virus strains with reduced susceptibility to NAIs may appear with the use of these antivirals or even naturally. We therefore screened the neuraminidase (NA) sequences of seasonal influenza virus A(H1N1), A(H1N1)pdm09, A(H3N2), and influenza B virus strains identified in Thailand for the presence of substitutions previously reported to reduce susceptibility to NAIs. We initially examined oseltamivir resistance (characterized by the H275Y mutation in the NA gene) in 485 A(H1N1)pdm09 strains circulating in Thailand and found that 0.82% (4/485) had this substitution. To further evaluate the evolution of the NA gene, we also randomly selected 98 A(H1N1)pdm09, 158 A(H3N2), and 69 influenza B virus strains for NA gene amplification and sequencing, which revealed various amino acid mutations in the active site of the NA protein previously shown to be associated with reduced susceptibility to NAIs. Phylogenetic analysis of the influenza virus strains from this study and elsewhere around the world, together with the estimations of nucleotide substitution rates and selection pressure, and the predictions of B-cell epitopes and N-linked glycosylation sites all provided evidence for the ongoing evolution of NA. The overall rates of NA evolution for influenza A viruses were higher than for influenza B virus at the nucleotide level, although influenza B virus possessed more genealogical diversity than that of influenza A viruses. The continual surveillance of the antigenic changes associated with the NA protein will not only contribute to the influenza virus database but may also provide a better understanding of selection pressure exerted by antiviral use.

## Introduction

The World Health Organization (WHO) have highlighted that the influenza virus causes approximately 3 to 5 million cases of influenza every year, which contribute to 250,000 to 500,000 deaths and 200,000 hospitalizations annually [1]. Since 1977, the seasonal influenza A(H1N1), A(H1N1)pdm09, A(H3N2), and the influenza B virus have co-circulated globally [2–3]. Antivirals against influenza virus are effective for the prevention of these viral infections and have been shown to reduce the duration of infection, the severity of illness, and mortality [4–6].

Hemagglutinin (HA) and neuraminidase (NA) are the two major surface glycoproteins of the influenza virus. HA is a homo-trimeric type I integral membrane protein that plays a role in the attachment of the virion to the host receptors and is targeted by the host immune response [7–8]. NA is a tetrameric type II integral membrane protein with sialidase activity responsible for releasing the newly produced viral particles [8–9].

Current treatment for influenza virus infection is limited to a single class of antivirals, namely neuraminidase inhibitors (NAIs) [10–11]. Although the structure of the catalytic and antigenic sites of NA protein of the influenza virus was identified in 1983 [12], the continual evolution of the NA gene has resulted from nucleotide substitutions, insertions, and deletions [13]. The relatively low fidelity of the influenza virus RNA polymerase contributes to the high rate of replication errors, which occur at approximately 1 in 10^4^ bases per replication cycle [14]. Thus, each round of replication leads to a population with more variants [13]. The resulting changes in the NA protein can modify the virus so that it can escape the host’s immune system or be resistant to antiviral drugs and persist in the human population [10, 15].

Currently, clinically approved NAIs include oseltamivir, zanamivir, peramivir, and laninamivir [16–17]. However, reports of emerging resistance to NAIs among some circulating strains of influenza virus have appeared [18–24]. Therefore, careful surveillance of the genetic variability of the NA gene may provide important insight into the evolution of the influenza virus. In this study, we examined for the presence of NA substitutions associated with reduced susceptibility to NAIs among influenza A and B viruses identified in Thailand. We further identified the B-cell epitopes and the potential N-linked glycosylation sites of the NA proteins, and to determine the evolutionary dynamics of the NA genes of strains of seasonal A(H1N1), A(H1N1)pdm09, A(H3N2), and influenza B viruses circulated in Thailand. The findings will aid in the understanding of the evolution of the viruses and provide surveillance data on NAI-resistant influenza virus strains. The antiviral susceptibility monitoring is important to provide the information about pandemic preparedness strategies for appropriate outbreak treatment and control.

## Materials and methods

### Ethical consideration

Respiratory samples were collected from patients with influenza-like illness (ILI) and analyzed at the Center of Excellence in Clinical Virology at King Chulalongkorn Memorial Hospital as part of the routine influenza surveillance program. The study protocol was approved by the Institutional Review Board (IRB) of the Faculty of Medicine at Chulalongkorn University (IRB No. 581/58). The study was conducted in accordance with the Declaration of Helsinki, and the IRB waived the need for consent because the samples were de-identified and anonymous. All the samples were acquired with permission from the Director of King Chulalongkorn Memorial Hospital.

### Clinical samples

The matrix (M) and HA genes PCR positive respiratory samples (nasal and nasopharyngeal swabs and aspirates, throat swabs, and bronchoalveolar lavage) for influenza A(H1N1)pdm09, A(H3N2), and B viruses between 2010 and 2015 in Thailand were used from the current study [25]. From 707 samples found to be positive for A(H1N1)pdm09 between November 2010 and December 2015, a total of 485 were randomly chosen for analysis of oseltamivir resistance (H275Y mutation in the NA gene) using real-time reverse transcription polymerase chain reaction (RT-PCR) for the NA gene and direct sequencing. In addition, influenza virus-positive respiratory samples [A(H1N1)pdm09 (N = 98), A(H3N2) (N = 158), and influenza B virus (N = 69)] collected by the Center of Excellence in Clinical Virology during the 2012 to 2015 influenza seasons were randomly selected for NA gene amplification and sequencing from original materials without prior virus isolation.

### NA amplification and sequence analysis

The NA segment of strains of A(H3N2), A(H1N1)pdm09, and influenza B virus were amplified using conventional PCR assays according to previously reported protocols and primer sets [26–28]. Briefly, viral RNA was extracted from the respiratory samples using a commercial viral nucleic acid extraction kit (GeneAll Biotechnology, Seoul, Korea). Thereafter, cDNA was synthesized using the ImProm-II Reverse Transcription System (Promega, Madison, WI) and 1 μM of either universal primers (Uni12 primer 5’-AGCAAAAGCAGG-3’) for influenza A virus or (FluB primer 5’-AGCAGAAGCA-3’) for influenza B virus. The PCR master mix contained 5 μl PRIME MasterMix (5Prime, Hamburg, Germany), 0.25 mM of MgCl_2_, 0.5 μM each of forward and reverse primers, 2 μl of cDNA template, and nuclease-free water to a final volume of 25 μl. Amplification was performed in a thermal cycler under the following conditions: initial denaturation at 94°C for 3 minutes, 40 cycles of 30 seconds of denaturation at 94°C, 30 seconds of primer annealing at 55°C, 90 seconds of extension at 72°C, and final extension for 7 minutes at 72°C. The PCR products were visualized on 2% agarose gel stained with ethidium bromide. The expected PCR products were purified using an Expin Combo GP kit (GeneAll Biotechnology, Seoul, Korea) according to the manufacturer’s protocol. The NA sequencing was performed by First BASE Laboratories (Selangor, Malaysia).

The NA sequences of A(H3N2) (KP336040 to KP336156 and KX151186 to KX151226), A(H1N1)pdm09 (KX151227 to KX151324), and influenza B virus (KX151325 to KX151393) in the present study were deposited in GenBank (S1 Table). The accession numbers associated with the 753 NA sequences of A(H3N2) (1998–2015), A(H1N1)pdm09 (2009–2015), seasonal A(H1N1) (2004–2009) and influenza B virus (1990–2015) circulated in Thailand, of which 118 were NA sequences of seasonal A(H1N1) reference strains obtained from the databases of GenBank and the Global Initiative on Sharing All Influenza Data (GISAID) (S1 Table). We analyzed for the presence of NA substitutions associated with either NAI-resistant genotype in different NA subtypes (E119V/I/A/G, H274Y, R292K, and N294S: N2 numbering) or reduced susceptibility genotype to NAIs (Q136K, D151E/V/D, D198N/G/E/Y, I222V/T/K/R/M, S246N, E276D, and R371K: N2 numbering) among influenza A and B viruses.

### Detection of oseltamivir resistance in A(H1N1)pdm09 strains

A real-time RT-PCR assay for detection of oseltamivir-resistant A(H1N1)pdm09 (H275Y) strains has been published previously [29]. Briefly, 10 μl reaction mixture comprised 3 μl of RNA, 0.75 μM of each primer, 0.25 μM of each probe, 2.5 mM of MgSO_4_, 5 μl of 2X reaction buffer (Invitrogen, Carlsbad, CA), 0.2 μl of SuperScript III RT/ Platinum Taq High Fidelity Enzyme Mix (Invitrogen, Carlsbad, CA), and water. Amplification and data analysis were performed using the LightCycler 480 Real-Time PCR System (Roche Diagnostics, Mannheim, Germany) under the following condition: reverse transcription at 50°C for 45 minutes, initial denaturation at 95°C for 10 minutes, 50 cycles of 15 seconds of denaturation at 95°C, and 40 seconds of annealing and extension at 60°C. The threshold cycle (C_t_) values obtained from both FAM and JOE channels were used to calculate the relative quantities by employing the delta-delta C_t_ (ΔΔC_t_) method.

### Phylogenetic analysis

The nucleotide sequences of the coding regions of NA genes of seasonal A(H1N1) (1–1410), A(H1N1)pdm09 (1–1407), A(H3N2) (1–1407) and influenza B virus (8–1408) viruses were aligned using ClustalX version 2.1 [30]. The Bayesian Information Criterion (BIC) and the maximum-likelihood value indicated the best-fit model for the seasonal A(H1N1) (T92+G+I), A(H1N1)pdm09 (T92+G), A(H3N2) (GTR+G), and influenza B virus (T92+G) datasets [31]. The phylogenetic trees of the NA nucleotide sequences were constructed using Molecular Evolutionary Genetics Analysis (MEGA) version 6.06 [32] employing maximum-likelihood tree with 1,000 bootstrap replicates. Bootstrap values >70% were shown.

### Estimation of nucleotide substitution rates

The best nucleotide substitution model was estimated using jModelTest software version 2.1.3 [33] and a GTR model with Gamma-distributed rate variation among sites was selected. The overall rates of evolutionary change (substitutions/site/year) and the relative genetic diversity of the NA genes were determined using the Bayesian Markov Chain Monte Carlo (MCMC) method implemented in Bayesian Evolutionary Analysis Sampling Trees (BEAST) software version 1.8.2 [34].

Sequence dataset comprised seasonal A(H1N1) (N = 144), A(H1N1)pdm09 (N = 306), A(H3N2) (N = 373), and influenza B virus (N = 255) from 989 viruses circulating in Thailand and 89 closely related reference strains from around the world with known sampling dates. For each analysis, a strict clock and uncorrelated log-normal relaxed clock model were both used under a GTR+G or GTR+G+I substitution model. The Bayesian skylines (BSP) and Gaussian Markov Random Field (GMRF) were used as coalescent prior. Two independent Bayesian MCMC analyses were run for 100 million states, sampling every 1,000 states. The convergence and effective sample sizes (ESSs) were assessed using Tracer software version 1.6, and ESSs values of ≥200 were accepted. The maximum clade credibility (MCC) tree was generated using TreeAnnotator software version 1.8.2 with 10% burn-in, and Figtree software version 1.4.2 was used to visualize the annotated trees. The uncertainty in each parameter estimate was reported using 95% highest posterior density (HPD) intervals.

### Estimation of selection pressure

In order to identify the selection pressure associated with the NA gene, the ratio of nonsynonymous substitutions (d*N*) and synonymous substitutions (d*S*) was estimated (d*N*/d*S*, defined as ω) using the single-likelihood ancestor counting (SLAC) method. Codon by codon basis of positively selected sites was further identified in HYPHY software [35] using the SLAC, the fixed effects likelihood (FEL), and the mixed effects model of evolution (MEME) methods. All the analyses were performed using the Datamonkey online tool. For each method, each codon with a *Ρ*-value of < 0.1 was determined to be a positively selected site.

### Prediction of potential N-linked glycosylation sites

The N-linked glycosylation sites were predicted using the NetNGlyc 1.0 Server [36]. For each of the NA proteins, the consensus sequences associated with N-linked glycosylation sites in proteins (i.e., amino acids Asn-X-Ser/Thr, where X is any amino acid except for Asp or Pro) were examined using the artificial neural networks on the server. A threshold value of >0.5 for the mean potential score was treated as indicative of glycosylation.

### Prediction of B-cell epitopes

In order to examine how the amino acid mutations found in the NA proteins of the 989 seasonal influenza A and B viruses circulated in Thailand may affect the antigenic properties of the NA protein, we predicted B-cell epitopes in the NA proteins using the BepiPred 1.0 Server. This server predicts the location of linear B-cell epitopes using a combination of a hidden Markov model and the propensity scale method [37]. The alignment of the NA protein sequences was submitted to the server and residues were annotated as being a part of linear B-cell epitopes when the score was above a particular threshold. The score threshold of 0.35 indicated the presence of an epitope, which corresponds to a sensitivity of 0.49 and a specificity of 0.75.

## Results

### Genotypic analysis of the neuraminidase inhibitors resistance among influenza A and B viruses

In the years after the influenza A virus pandemic of 2010, 485 samples tested positive for A(H1N1)pdm09 of which 4 (0.82%) were oseltamivir-resistant (H275Y) strains (S1 Fig and S2 Table). We next screened NA sequences for the presence of substitutions previously reported to reduce susceptibility to NAIs by the WHO expert working group on surveillance of influenza antiviral susceptibility (AVWG) 2014 (19–22; 24). Numbering is based on an alignment of NAs from the following reference strains: A/Brisbane/59/2007 (H1N1), A/California/7/2009(H1N1) pdm09, A/Perth/16/2009 (H3N2), and B/Yamanashi/166/1998. The NA sequences of seasonal A(H1N1) viruses circulated from 2004 to 2009 in Thailand showed that the resistance rate to oseltamivir was 17% (20/118) (Table 1). Between 2009 and 2015, 6.4% of viruses circulating in Thailand of A(H1N1)pdm09 were H275Y strains, whereas, 1.4% had an S247N mutation. The A(H3N2) viruses circulating in Thailand had amino acid substitutions at positions D151N/G (1.7%) and I222T/V (1.2%), which are in the active site of the NA protein and may affect enzyme activity. The NB sequences of influenza B virus circulated in Thailand between 1990 and 2015 had an amino acid mutation at position D197N (0.4%), and an amino acid substitution at position A395E (1.7%) was also found. In addition, we found that the NA protein in influenza B was mutated at positions A245S (0.4%), K360R (0.9%), and A395V/T/D/S (12.5%).

**Table 1:** The frequency of NA amino acid substitutions associated with reduced inhibition by NAIs among influenza A and B viruses circulated in Thailand in 1998–2015.

Type/Subtype (year of circulation)	Amino acid substitution ^a^	No. mutant viruses/no. total (%) ^b^
A(H1N1) (2004–2009)	H275Y	20/118 (16.95)
A(H1N1)pdm09 (2009–2015)	S247N	4/296 (1.35)
	H275Y	19/296 (6.42)
Influenza B virus (1990–2015)	D197N	1/232 (0.43)
	A395E	4/232 (1.72)

^a^ Numbering is based on an alignment of NAs from the following reference strains: A/Brisbane/59/2007 (H1N1), A/California/7/2009 (H1N1)pdm09, A/Perth/16/2009 (H3N2), and B/Yamanashi/166/1998.

^b^ The total number of NA sequences of influenza A and B viruses identified in Thailand and deposited on the NCBI and GISAID.

### Phylogenetic analysis

To examine the genetic variability of the NA genes of influenza A and B viruses circulating in Thailand, we conducted a phylogenetic analysis of NA of 118 seasonal A(H1N1), 296 A(H1N1)pdm09, 343 A(H3N2), and 232 influenza B viruses. The designation of the clades for NA phylogenetic trees of influenza viruses in the present study is based on WHO influenza center London [38]. In addition, we screened for the presence of NA molecular markers associated with reduced NAIs susceptibility.

**Fig 1:**
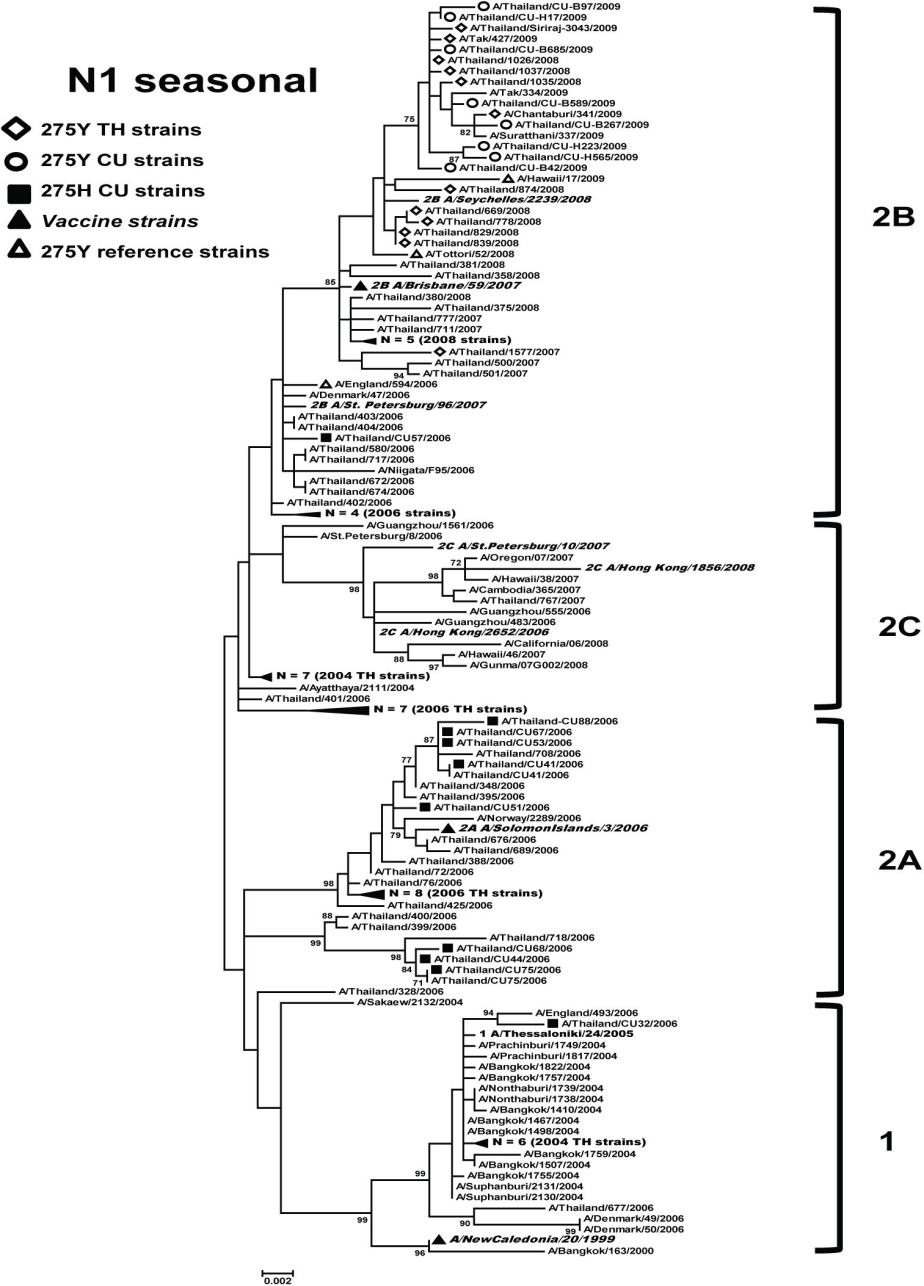
Maximum-likelihood phylogenetic tree of the NA gene of seasonal A(H1N1) influenza viruses circulated in Thailand. NA sequences from 118 A(H1N1) strains circulated in Thailand between 2000 and 2009 were compared to the reference strains of known clades reported by WHO Influenza Center London (bolded) and the southern hemisphere vaccine strains recommended by WHO (denoted as ▲). Bootstrap values >70% are shown at the branch nodes. Scale bar represents approximately 0.2% nucleotide difference between close relatives.

The seasonal A(H1N1) viruses circulating in Thailand from 2004 to 2006 were oseltamivir-susceptible and belonged to clades 1, 2A, and 2C (Fig 1). However, the strains from 2007 to 2009 were mostly oseltamivir-resistant (54.05%, 20/37) and belonged to clade 2B. Most of these oseltamivir-resistant viruses (95%, 19/20) from 2007 to 2009 had a D354G mutation in the coding region when compared with the reference strain (A/Brisbane/59/2007).

**Fig 2:**
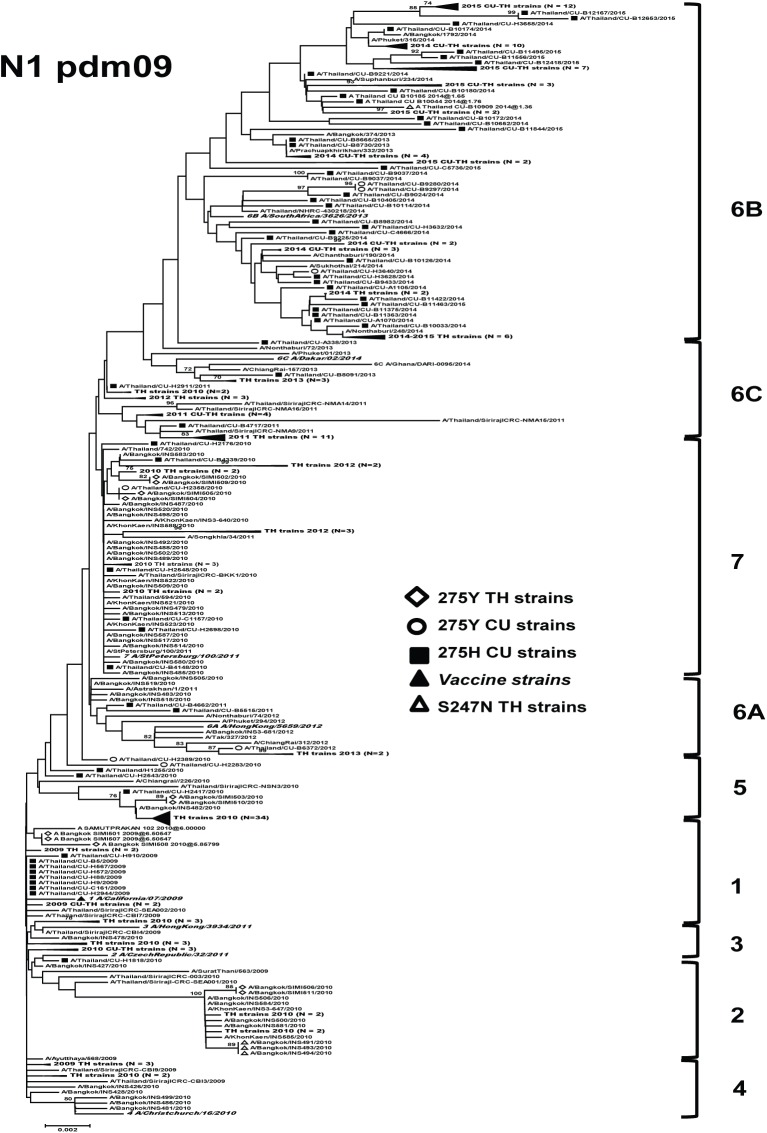
Maximum-likelihood phylogenetic tree of the NA gene of A(H1N1)pdm09 influenza viruses circulated in Thailand. NA sequences from 296 A(H1N1)pdm09 strains circulated in Thailand between 2009 and 2015 were compared to the reference strains of known clades reported by WHO Influenza Center London (bolded) and the southern hemisphere vaccine strains recommended by WHO (denoted as ▲). Bootstrap values >70% are shown at the branch nodes. Scale bar represents approximately 0.2% nucleotide difference between close relatives.

For A(H1N1)pdm09, strains with mutation S247N circulated in 2010 belonged to clade 2 (Fig 2). They possessed amino acid mutations at positions N248D, I389V, and V394I compared with the reference (strain A/California/07/2009) (Table 2). In contrast, the H275Y oseltamivir-resistant strains that were circulated in other years were clustered separately: clade 1 (2009 season), clades 2, 5, and 7 (2010 season), clade 6A (2012 season), and clade 6B (2014 season). Most of the H275Y oseltamivir-resistant strains had the additional NA mutations at positions V106I, V241I, N248D, and N369K.

**Table 2:** NA amino acid substitutions associated with reduced inhibition by NAIs among influenza A and B viruses circulated in Thailand.

Position	Reference strain ^a^	Observed residues (number of strains)	Major^b^ (%)
**A(H1N1) seasonal (H275Y: 20 sequences)**			
221–222	KQ	KQ (16), KR (2), EQ (2)	80
354	D	G (19), D (1)	95
**A(H1N1)pdm09 (S247N: 4 sequences)**			
248	N	D (4)	100
389	I	V (3), I (1)	75
394	V	V (3), I (1)	75
**A(H1N1)pdm09 (H275Y: 19 sequences)**			
43–44	QN	QS (9), QN (8), KN (2)	47.4
106	V	I (16), V (3)	84.2
241	V	I (12), V (7)	63.2
248	N	D (19)	100
369	N	K (13), N (6)	68.4
**Influenza B virus (D197N and A395E: 5 sequences)**			
41–42	SP	SP (4), SQ (1)	80
49–50	TM	TM (4), IM (1)	80
125	N	N (4), T (1)	80
198	N	N (4), S (1)	80
204	V	V (4), I (1)	80
219–220	NK	NK (4), KN (1)	80
320	D	D (4), E(1)	80
358	E	E (4), A (1)	80
378	G	E (4), G (1)	80
389	A	A (4), T (1)	80
395–396	AF	EF (4), AL (1)	80
404	K	K (4), E (1)	80
463	D	N (4), D (1)	80

^a^ Numbering is based on an alignment of NAs from the following reference strains: A/Brisbane/59/2007 (H1N1), A/California/7/2009 (H1N1)pdm09, A/Perth/16/2009 (H3N2), and B/Brisbane/60/2008.

^**b**^ Denotes the proportion of the most commonly observed residues for a given position.

**Fig 3:**
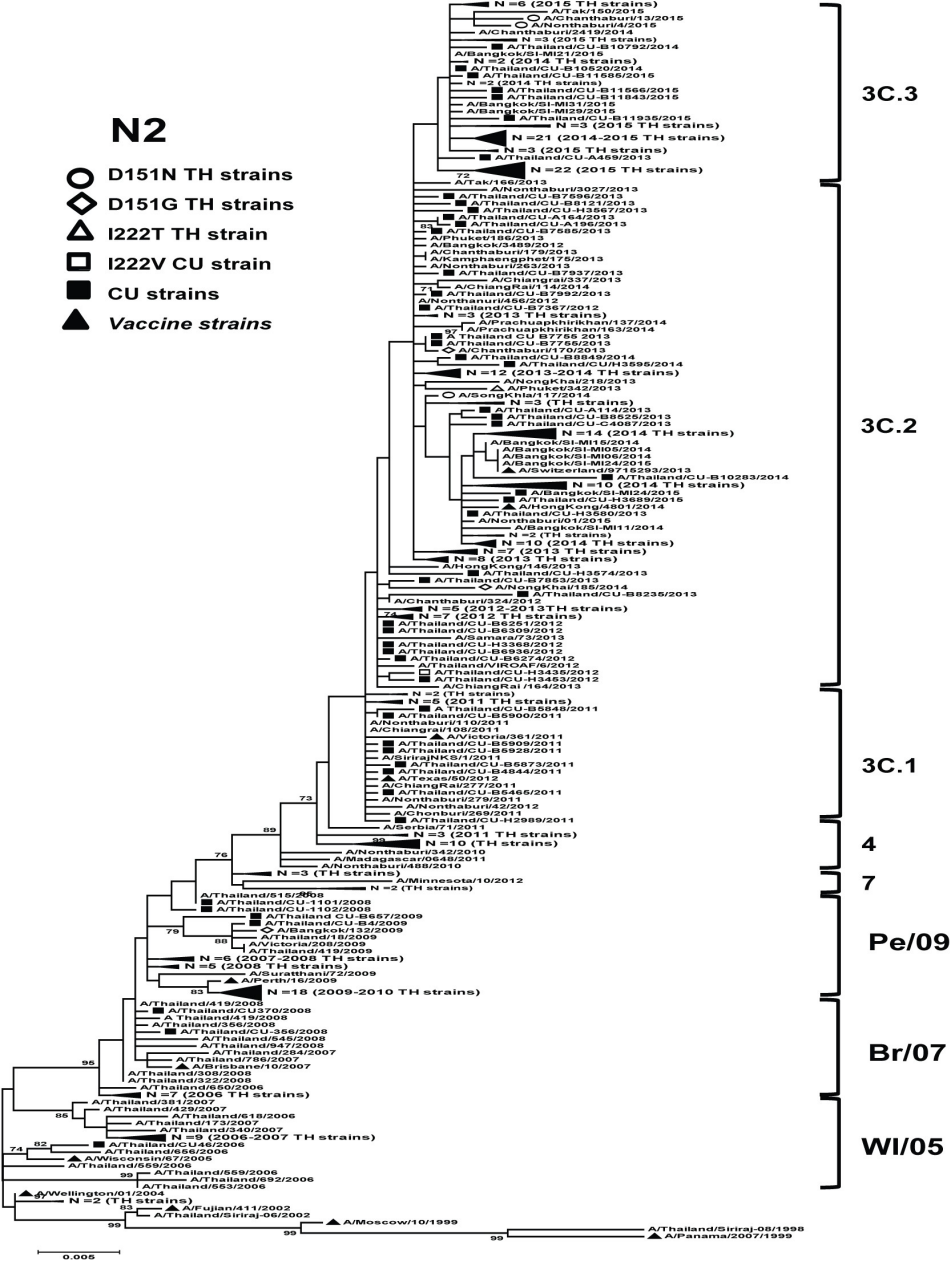
Maximum-likelihood phylogenetic tree of the NA gene of A(H3N2) influenza viruses circulated in Thailand. NA sequences from 343 A(H3N2) strains circulated in Thailand between 2002 and 2015 were compared to the southern hemisphere vaccine strains recommended by WHO (denoted as ▲). Bootstrap values >70% are shown at the branch nodes. Scale bar represents approximately 0.2% nucleotide difference between close relatives. Pe/09 denotes A/Perth/16/2009, Br/07 denotes A/Brisbane/10/2007, and WI/05 denotes A/Wisconsin/67/2005.

For A(H3N2), the strains demonstrated a typical ladder-like gradual evolution, with the replacement of old strains by newer ones (Fig 3). The strains with a I222T/V mutation were grouped into clade 3C.2; these two strains maintained the NA gene signature amino acid substitutions L81P, D93G, S367N, K369T, N402D, and I464L. The strains with a D151N/G mutation were clustered separately in clades 3C.2 and 3C.3. Most of these strains (83.3%) had amino acid mutations at positions L81P, D93G, S367N, K369T, N402D, and I464L.

**Fig 4:**
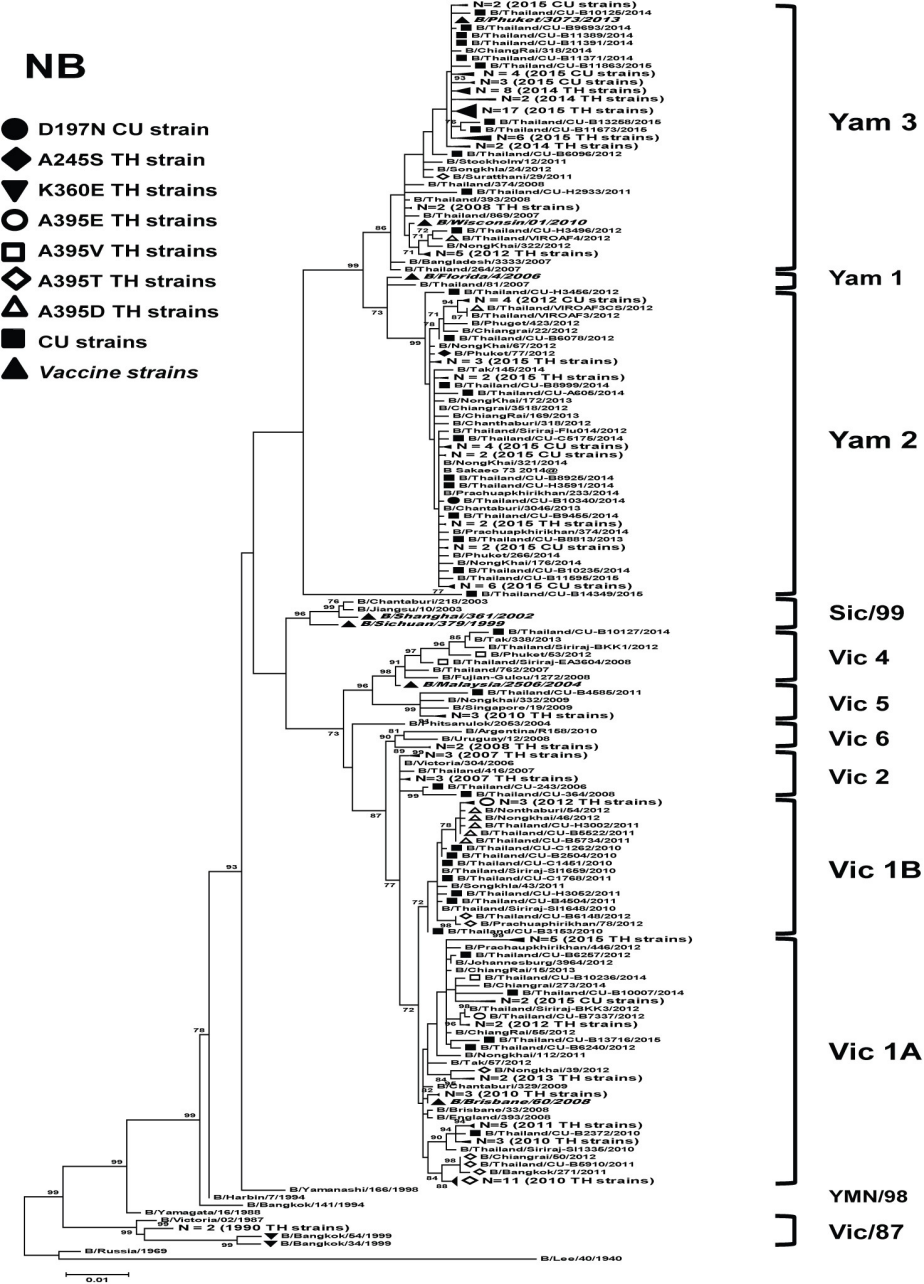
Maximum-likelihood phylogenetic tree of the NA gene of influenza B viruses circulated in Thailand. NA sequences from 232 influenza B strains circulated in Thailand between 1990 and 2015 were compared to the southern hemisphere vaccine strains recommended by WHO (denoted as ▲). Bootstrap values >70% are shown at the branch nodes. Scale bar represents approximately 0.2% nucleotide difference between close relatives. Yam denotes Yamagata, Sic/99 denotes B/Sichuan/379/1999, Vic denotes Victoria, and YMN/98 denotes B/Yamanashi/166/1998.

For NA sequences of influenza B virus, the A395E NAI-resistant strains belonged to Victoria lineage (clade 1) (Fig 4). Only one D197N NAI-resistant isolate was classified in Yamagata lineage (clade 2). Most of the strains with A395V/T/D substitutions were found in the Victoria lineages. The additional amino acid substitutions found in the NA proteins of suspected NAI-resistant of influenza B viruses from Thailand are summarized (Table 2).

### Evolutionary rate and ancestral time analysis

The NA phylogenic trees of influenza A(H1N1)pdm09 and A(H3N2) viruses showed according to the timescale analyses (S2 Fig). Based on the phylogenetic trees, posterior probabilities, marginal likelihoods, Bayes factor and convergence in terms of ESS values, the best-fit models for the NA gene of influenza A(H1N1)pdm09 and A(H3N2) data sets were the strict clock models with constant population size. Based on these models, the rates of nucleotide substitution for the NA genes of A(H1N1)pdm09 and A(H3N2) were estimated to be 3.49 × 10^−3^ substitutions/site/year (95% highest posterior density, HPD: 3.12 × 10^−3^–3.88 × 10^−3^) and 3.18 × 10^−3^ substitutions/site/year (95% HPD: 2.64 × 10^−3^–3.72 × 10^−3^), respectively. Estimating time to the most recent common ancestor (TMRCA) for (H3N2) gene and for the A(H1N1)pdm09 gene were 49.38 years (95% HPD: 48.84–49.93) and 9.52 years (95% HPD: 8.70–10.45), respectively.

In contrast, the best-fit models for the NA genes of influenza seasonal A(H1N1) and influenza B virus data sets were relaxed uncorrelated log-normal molecular clock models with constant population size (Table 3). Based on the timescale analyses, NA phylogenies of seasonal A(H1N1) and influenza B virus are shown (S2 Fig). The rates of nucleotide substitutions of NA genes for the seasonal A(H1N1) and influenza B virus genes were estimated to be 6.26 × 10^−3^ substitutions/site/year (95% HPD: 3.79 × 10^−3^–9.15 × 10^−3^) and 1.61 × 10^−3^ substitutions/site/year (95% HPD: 1.14 × 10^−3^–1.70 × 10^−3^), respectively. The mean of the root age for the seasonal A(H1N1) gene and for the influenza B virus gene were estimated to be 14.55 years (95% HPD: 13.85–15.62) and 108.13 years (95% HPD: 89.31–133.14), respectively.

**Table 3:** Estimating time to the most recent common ancestor (TMRCA) and nucleotide substitution rate of the NA genes among influenza A and B viruses circulating in Thailand and globally.

Types/subtypes	Clock Model	TMRCA of root height		Nucleotide substitution rate	
				× 10^−3^ (subs/site/year)	
		Mean	95%HPD Interval	Mean	95%HPD Interval
A(H1N1)seasonal	Uncorrelated lognormal relaxed clock	14.55	13.85–15.62	6.26	3.79–9.15
A(H1N1)pdm09	Strict clock	9.52	8.70–10.45	3.18	2.64–3.72
A(H3N2)	Strict clock	49.38	48.84–49.93	3.49	3.12–3.88
B	Uncorrelated lognormal relaxed clock	108.13	89.31–133.14	1.61	1.14–1.70

### Selection pressure analysis

We investigated the selection pressure in NA gene of influenza A and B viruses for understanding its evolutionary dynamics. The positive selection in NA genes indicates to the viral adaptation to the new human host, potential evasion of the host immune system, and balancing functionality between HA and NA. The overall global ω values of the A(H1N1)pdm09 (0.31) and influenza B virus (0.30) genes were higher than those of the seasonal A(H1N1) (0.26) and A(H3N2) (0.24) genes (Table 4). Although overall positive selection was not present, specific sites of positive selection were found (Table 4). A statistically significant result in each method was defined (S4 Table). The results of the MEME analyses indicated that the number of positively selected sites for the A(H3N2) and influenza B virus genes were higher than for both the A(H1N1)pdm09 and seasonal A(H1N1) genes.

**Table 4:** The positively selected sites from selection pressure analysis of the NA gene of Thai seasonal influenza isolates.

Types/Subtypes	Global ω	No. of positively selected sites (codon position)		
	by SLAC	SLAC	FEL	MEME
A(H1N1)seasonal	0.26	3 (77, 222, 452)	5 (77, 222, 249, 344, 452)	7 (7, 77, 222, 249, 266, 344, 452)
A(H1N1)pdm09	0.31	1 (463)	7 (13, 34, 48, 188, 270, 451, 463)	6 (13, 34, 48, 188, 366, 463)
A(H3N2)	0.24	4 (93, 215, 401, 468)	9 (4, 43, 93, 141, 150, 215, 401, 464, 468)	12 (4, 43, 93, 141, 181, 215, 267, 271, 401, 402, 464, 468)
B	0.30	3 (73, 395, 404)	9 (68, 73, 106, 220, 248, 358, 395, 404, 465)	13 (15, 27, 41, 51, 73, 106, 107, 219, 220, 358, 395, 404, 465)

Global ω denotes the ratio of nonsynonymous substitutions (dN) to synonymous substitutions (dS).

SLAC, single-likelihood ancestor counting; FEL, fixed effects likelihood; MEME, mixed effects model of evolution.

### Potential N-linked glycosylation sites

We predicted the potential N-linked glycosylation sites of the NA proteins of the 118 seasonal A(H1N1), 296 A(H1N1)pdm09, 343 A(H3N2), and 232 influenza B virus strains circulated in Thailand (Table 5). The NA proteins from seasonal A(H1N1) viruses had nine conserved N-linked glycosylation sites when compared with the reference strain (A/New Caledonia/20/1999), while those from A(H1N1)pdm09 viruses revealed eight glycosylation sites when compared with the reference strain (A/California/07/2009). Interestingly, for the viruses in sub-clades 6B and 6C, an N-linked glycosylation site was observed at position 42 of the A(H1N1)pdm09 protein and at position 386 in all the strains except for those in sub-clades 6A and 6B.

**Table 5:** Potential N-linked glycosylation sites of NA proteins in seasonal influenza strains circulating in Thailand.

NA position	NA sequences	N-Gly Score^a^	Jury agreement^b^	N-Gly results^c^	Clade
**A(H1N1) seasonal (N = 118)**					
44	NHTG	0.671	(8/9)	+	All
58	NSTW	0.599	(8/9)	+	All
63	NHTY	0.560	(8/9)	+	All
70	NNTN	0.688	(8/9)	+	All
88	NSSL	0.767	(9/9)	+++	All
146	NGTV	0.687	(9/9)	++	All
235	NGSC	0.736	(9/9)	++	All
434	NTTI	0.610	(7/9)	+	All
455	NWSW	0.272	(9/9)	---	All
**A(H1N1)pdm09 (N = 296)**					
42	NQSQ	0.629	(8/9)	+	Only clade 6B, 6C
50	NQSV	0.554	(6/9)	+	All
58	NNTW	0.569	(6/9)	+	All
63	NQTY	0.681	(9/9)	++	All
68	NISN	0.737	(9/9)	++	All
88	NSSL	0.738	(9/9)	++	All
146	NGTI	0.660	(9/9)	++	All
235	NGSC	0.728	(9/9)	++	All
386	NFSI	0.294	(9/9)	---	All except clade 6A and 6B
**A(H3N2) (N = 343)**					
61	NITE	0.756	(9/9)	+++	All
70	NTTI	0.545	(6/9)	+	All
86	NWSK	0.604	(8/9)	+	All
93	NITG	0.685	(9/9)	++	Only clade WI/05
146	NDTV	0.642	(9/9)	++	All
200	NATA	0.361	(9/9)	--	All
234	NGTC	0.755	(9/9)	+++	All
329	NDSS	0.488	(5/9)	-	All except Br/07
367	NETS	0.540	(5/9)	+	3A, 3B, 3C.2, 3C.3, 4 and 6
402	NRSG	0.407	(6/9)	-	All except 3B, 3C and 4
**Influenza B virus (N = 232)**					
56	NASN	0.592	(7/9)	+	All
64	NRSA	0.685	(8/9)	+	All
144	NGTR	0.816	(9/9)	+++	All
284	NKTI	0.704	(9/9)	++	All
463	NMTL	0.425	(6/9)	-	Only clade Yam-3

^a^ The potential score is the averaged output of nine neural networks

^b^ The Jury agreement indicate how many of the nine networks support the prediction

^c^ The N-Glyc result indicates (+) potential N-glycosylated sites > 0.5 threshold, (++) potential N-glycosylated sites > 0.5 threshold and Jury agreement (9/9), (+++) potential N-glycosylated sites > 0.75 threshold and Jury agreement (9/9), (-) non-glycosylated sites < 0.5 threshold, (- -) non-glycosylated sites < 0.5 threshold and Jury agreement (9/9), and (- - -) non-glycosylated sites < 0.32 threshold.

Regarding the NA proteins of A(H3N2) viruses, seven N-linked glycosylation sites were conserved, except for the strains in clade A/Brisbane/10/2007, which had lost an N-linked glycosylation site at position 329. The viruses from Thailand in clade A/Wisconsin/67/2005 were found to have an N-linked glycosylation site at position 93. Surprisingly, the clade 3A, 3B, 3C.2, 3C.3, 4 and 6 of the A(H3N2) viruses circulating in Thailand that started emerging early in the 2011 season have an N-linked glycosylation site at position 367 but not at position 402 (3B, 3C and 4). For the NA proteins of influenza B viruses, four N-linked glycosylation sites were found, and the viruses in Yamagata lineage clade 3 also had an additional N-linked glycosylation site at position 463.

### Prediction of B-cell epitopes

For the seasonal A(H1N1), 24 residues were associated with the B-cell epitope profile. Significant antigenic variation between the clade 2C viruses from Thailand and the reference strain (A/New Caledonia/20/1999) was found at residues 99–101. Seasonal A(H1N1) strains in clade 2B did not have epitopes at residues 284–285, while strains in clade 2C did not have epitopes at residues 450–452 (S3 Fig.).

Meanwhile, 26 residues in the A(H1N1)pdm09 strains were found to be associated with the B-cell epitope profile in A/California/07/2009. Most of the viruses circulating in Thailand did not have epitopes at residues 102–104 and 110, except for the strains in sub-clades 6B and 6C. The strains in sub-clade 6B had epitopes at residues 224–228, whereas the strains in sub-clade 6A showed a loss of antigenicity at residues 385–388.

The A(H3N2) of the strain in clade A/Perth/16/2009 had B-cell epitopes at residues 301–303, 305, 307–308, and 310. The viruses from Thailand in clades 3B, 3C.1, 3C.2, 3C.3, and 4 had epitopes at residues 76–84.

The influenza B virus NA protein of Vic87 had 23 predicted B-cell epitope residues that are recognized by human antibodies. The epitopes of the NA protein of strains in Yamagata lineage clade 3 and Victoria lineage clade 5 differed from those of Vic87, as they had epitopes at residues 69–77 and 401–403, respectively. None of Yamagata strains identified in this study possessed epitopes at residues 48–53.

### Evolutionary dynamics of NA segments of seasonal influenza A and B viruses

**Fig 5:**
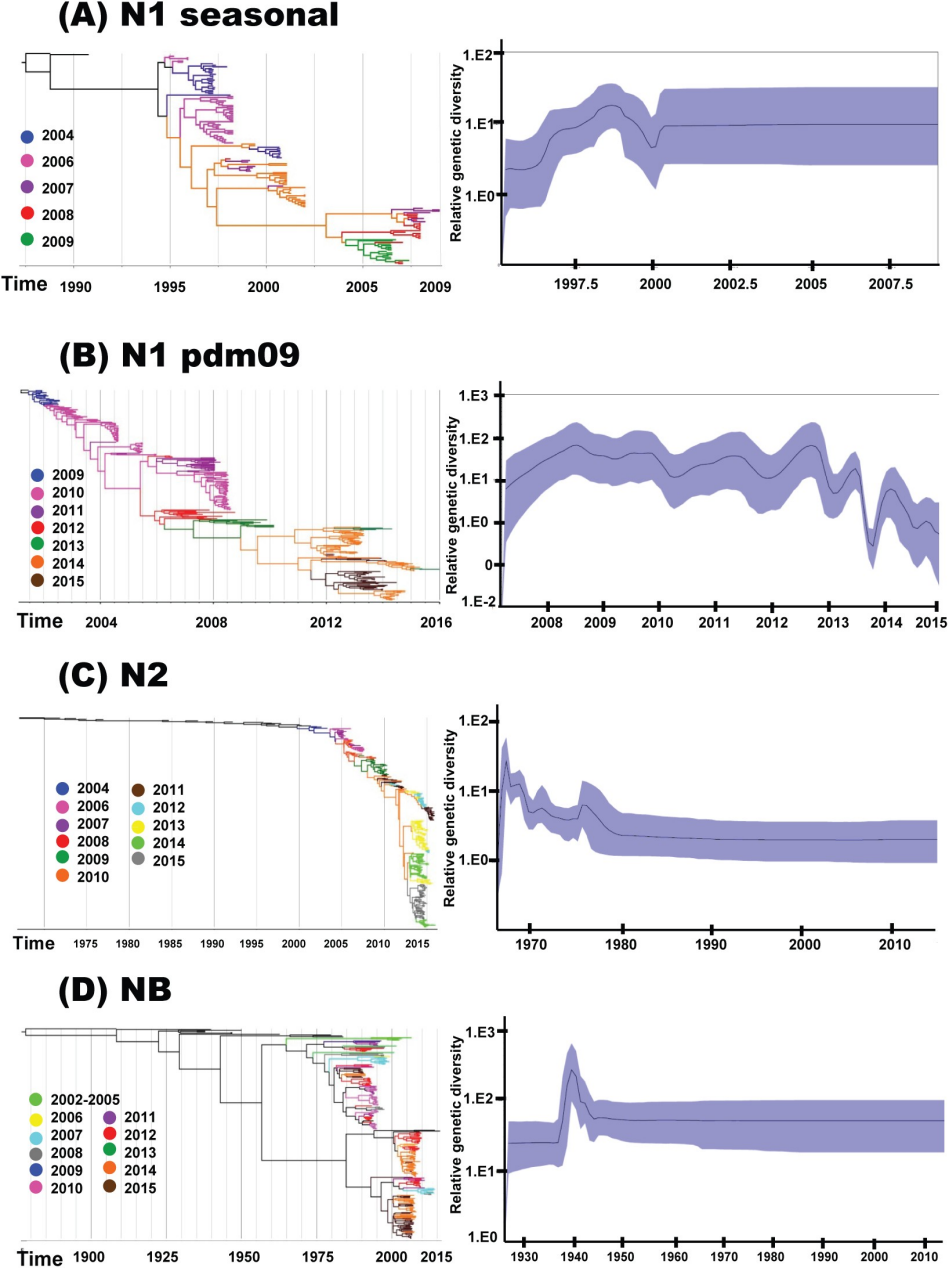
Temporal phylogeny and population dynamic diversity of seasonal influenza viruses circulating in Thailand. Evolution of the NA genes of (A) seasonal A(H1N1) (2004–2009), (B) A(H1N1)pdm09 (2009–2015), (C) A(H3N2) (2004–2015), and (D) influenza B virus (2002–2015). Phylogenetic trees were constructed using uncorrelated lognormal relaxed or strict clock model with branches colored by year of virus isolation and the relative genetic diversity of NA segments using the Gaussian Markov Random Field (GMRF) or Bayesian skyline (BS) model. Solid black lines in GMRF or BS plot denoted mean relative genetic diversity, while blue shade represents the 95% HPD intervals.

The phylogenetic trees of NA showed that each year and for each types/subtypes of influenza virus, strains from multiple clades were co-circulating (Fig 5). For A(H3N2), A(H1N1)pdm09, and seasonal A(H1N1) genes, the emergence of new clades/sub-clades occurred over short periods of time (with mean durations of 1.39, 1.97, and 2.35 years, respectively). In contrast, the NB genes of influenza B viruses persisted unchanged over 3 and 8 seasons for the Victoria and Yamagata lineages, respectively (with mean durations of 3.38 and 8.68 years).

Population dynamics of the NA genes of seasonal A(H1N1), A(H3N2), and influenza B viruses (Fig 5A, 5C and 5D) demonstrated the constant sizes of the populations during the period from 2002 to 2015. In contrast, the NA gene of the A(H1N1)pdm09 viral population indicated alternating of periods of exponential growth and oscillating patterns of relative genetic diversity after the early pandemic spread of the A(H1N1)pdm09 strain (Fig 5B). This pattern indicated increases in genetic diversity followed by bottlenecks between the seasons. Interestingly, there was a marked decrease in the relative genetic diversity at the end of 2012 until the mid-2014 season, with periods during which the numbers of A(H1N1)pdm09 infections decreased (S1 Fig), while the number of A(H3N2) infections increased. Overall, the relative genetic diversity of the NA gene of A(H1N1)pdm09 during the 2009–2011 seasons and of influenza B virus was higher than that of seasonal A(H1N1), post-pandemic A(H1N1)pdm09, and A(H3N2).

## Discussion

Strains of A(H1N1)pdm09 have co-circulated with A(H3N2) and influenza B viruses to a varying extent during most influenza seasons. Published surveillance data have demonstrated that the prevalence of oseltamivir resistance characterized by the mutation at residue H275Y in NA gene of A(H1N1)pdm09 viruses is still low (<3%) [16, 39–40]. In Thailand, during the second and third waves of the A(H1N1)pdm09 pandemic from January 2010 to October 2010, the prevalence of oseltamivir-resistant strains was 0.44% [26]. The present study showed that the prevalence of oseltamivir-resistant strains during the post-pandemic period was slightly higher than that during the pandemic (0.82%; 4/485). Based on the ΔΔC_t_ method calculation using real-time RT-PCR, a mix of the wild-type susceptible strain (275H) and resistant strain (275Y) was detected in all the H275Y-positive samples and the relative quantities of RNA of the latter virus were higher than those of the former virus. This finding indicates that the resistant strains have potentially advantageous mutations that allow them to become dominant when the antiviral is used [41]. Furthermore, the majority of individuals infected with the H275Y variants were in “at risk” groups (i.e. individuals with hematologic cancers, allergic rhinitis, and the elderly) who are more likely to have the strain due to their immunocompromised status [42].

Hurt and colleagues found that a novel influenza A(H1N1)pdm09 variant with mildly reduced oseltamivir and zanamivir sensitivity has been detected in more than 10% of community specimens in Singapore and more than 30% of samples from northern Australia [22]. In Thailand, the prevalence of this S247N mutation in A(H1N1)pdm09 was lower (1.35%) than in Australia and Singapore, which might be due to sampling size bias. In the NA gene of the A(H1N1)pdm09 circulated in Thailand, the H275Y strains had permissive mutations (define as mutations that allowed the virus to tolerate subsequent occurrences of H275Y) at residues V241I (63%) and N369K (68%). It has been suggested that these variants might be able to replicate and transmitted more efficiently [43].

The earliest reports of the detection of oseltamivir-resistant seasonal A(H1N1) were in November 2007 from France, Norway, and the United Kingdom [18]. In the present study, the proportion of H275Y strains during the period from 2007 to 2009 was 54.05% (20/37); which is consistent with findings from other studies [18, 44–45]. The H275Y mutation can confer cross-resistance to peramivir, but susceptibility to zanamivir and laninamivir is not significantly affected [16]. As noted in a previous study, the H275Y seasonal A(H1N1) strains with reduced viral fitness had permissive substitutions at V234M (100%), R222Q (90%), K329E (100%), and D344N (100%), and compensatory mutation D354G (95%), which maintained the functionality of the NA protein [46].

The surveillance data on seasonal influenza reveal that the incidence of oseltamivir and zanamivir resistance was low in A(H3N2) strains (1–3%) and rare (<1%) in influenza B virus strains [47–48]. Drug resistance characterized by E119V or R292K mutation (according to N2 numbering) was not observed in the present study. However, the catalytic site mutation (D151N/G) and framework mutation (I222T/V) of the NA protein were found. The NA protein of influenza B virus in Thailand between 1990 and 2015 showed D197N mutation, which is associated with reduced susceptibility to oseltamivir and zanamivir [19]. In influenza B virus, the A395E substitution located outside the active site of NA is associated with reduced susceptibility to oseltamivir and peramivir [24]. Interestingly, we found a high frequency of NA mutations A395V/T/D/S in the influenza B viruses circulating in Thailand. Mutations at the active site of the NA protein in strains of both A(H3N2) and influenza B virus may affect the half maximal inhibitory concentration (IC_50_) of the NAIs. In present study, the antiviral susceptibility of the viruses with D151N/G, I222T/V, and A395E/V/T/D/S NA substitutions cannot be concluded by genotypic methods. Therefore, the phenotypic assay to confirm the NAI resistance profile of these NA substitutions should be needed.

The continual genetic drift of influenza virus resulting from cumulative mutations may introduce novel N-linked glycosylation sites and alter antigenic epitopes of progeny viruses. The loss or gain of glycosylation may also change the NA protein’s substrate specificity profiles and correct molecule folding, which affects the NA protein’s enzymatic activity [49]. Moreover, glycosylation of the NA protein may change the antigenic properties of the virus and glycosylation may affect its pathogenicity [50]. Therefore, potential N-linked glycosylation sites in the NA proteins of seasonal influenza A and B circulated in Thailand were assessed.

We observed that the overall rate of nucleotide substitution was higher for the NA gene of the seasonal A(H1N1) viruses than for the NA genes of the A(H1N1)pdm09, A(H3N2), and influenza B viruses similar to previous studies. For example, A(H3N2) viruses collected between 1968 and 2011 worldwide (N = 286) had a mean nucleotide substitution rate of 3.27 × 10^−3^ substitutions/site/year [51]. In North America, studies have reported rates of (3.11–12.50) x 10^−3^ [52] and 5.41 x 10^−3^ [53]. For influenza B viruses, the nucleotide substitution rate of the NA gene in the present study is comparable to those reported in Australia and New Zealand (mean nucleotide substitution rate of 2.04 × 10^−3^ substitutions/site/year for the Victoria lineage and 2.25 × 10^−3^ substitutions/site/year for the Yamagata lineage) [54] and Malaysia (2.5–3.4 x 10^−3^) [55]. In contrast, the mean nucleotide substitution rate of the NA gene of A(H1N1)pdm09 in the present study was slightly lower than a previous study of 5.21 × 10^−3^ substitutions/site/year [3] and 5.27 x 10^−3^ [56], which may be attributed to the study sample size and sequence diversity of the strains.

The mean of the global ω values was similar for all the NA genes of the strains of seasonal influenza virus in Thailand. Since the majority of the NA protein residues of influenza A and B viruses showed ω < 1, it suggests that the amino acids in this protein are under purifying selection. These findings are consistent with previous studies, which reported the overall ω values for the NA genes of 0.30 for seasonal A(H1N1), 0.32 for A(H1N1)pdm09, 0.29 for A(H3N2), and 0.20–0.31 for influenza B virus [3, 51, 54, 57]. The selection pressure of NA of A(H3N2) in German was 0.21, while in Taiwan it was 0.37 [58–59]. Moreover, the findings showed that NA gene had highest a number of amino acid residue under the positive selection. The large ω values observed for the NA of A(H1N1)pdm09 may indicate adaptation of the virus to its new human host or due to the observed imbalance in HA and NA functionality in some viruses [60], while the large ω values for the NA gene of influenza B may be due to broad NA-related immunity [61]. Moreover, the positively selected sites in the NA segment of influenza viruses in Thailand indicate the potential to evade the host immune response and NAIs [57]. The positively selected sites in the NA proteins [seasonal A(H1N1): 77, 249, 344, and 452; A(H1N1)pdm09: 48, 270, 366, 451, and 463; A(H3N2): 43, 93, 141, 150, 181, 267, 271, 401, 402, and 464; and influenza B virus: 51, 68, 73, 106, 107, 248, 395, 404, and 465] may represent the antigenic sites [12].

These defined epitopes were associated with the findings on B-cell epitope profiles predicted using the BepiPred 1.0 Server. In addition, the codon at residues 93 and 402 of the A(H3N2) protein (which were associated with the loss of an N-linked glycosylation site) were under positive selection pressure, reflecting the potential for the virus to evade the host immune system. The positively selected codon at residue 141 in A(H3N2) is linked to a calcium ion binding site, which plays an important role in stabilizing the conformation of the NA protein [62]. The mutation at codon 395 in influenza B virus conferred an increase in the IC_50_ of oseltamivir and peramivir and was under positive selection pressure. In addition, we found V106I and N248D mutations in the NA protein, which are located at the subunit interfaces and the primary calcium ion binding site that is associated with the stability of A(H1N1)pdm09 virus at a low pH [63].

The MCC tree from the Bayesian timescale phylogenetic analysis of the NA from seasonal influenza viruses circulating in Thailand is consistent with previous studies and highlights how circulating strains are continually drifting [3, 51, 54] whereby strains which are unable to evade the host immune system are eliminated [64]. The evolutionary patterns of the NA gene indicated a more rapid genetic drift for influenza A than influenza B virus due to higher nucleotide substitution rate, although there was more genetic diversity in influenza B than in influenza A virus. Therefore, there is an inverse relationship between the evolutionary rate and genealogical diversity, which is similar to the evolutionary pattern of the HA gene of seasonal influenza viruses across the globe [65].

In conclusion, this study found evidence for the ongoing evolution of the NA protein, including amino acid mutations in the active site that were associated with reduced susceptibility to NAIs and evolutionary parameters including the nucleotide substitution rate, the selection pressure, and the genetic diversity. Continual monitoring of the antigenic changes and evolutionary dynamics of the NA segments of the influenza virus will assist public health and clinical recommendations for antiviral use.

## Supporting information

S1 Fig Incidence of influenza A and B viruses identified from clinical samples between 2010 and 2015. (PDF)

S2 Fig A maximum clade credibility tree from Bayesian timescale phylogenetic analysis of NA genes of influenza A(H1N1)pdm09, seasonal A(H1N1), A(H3N2), and influenza B virus. (PDF)

S3 Fig Predicted B-cell epitopes of NA proteins of seasonal A(H1N1), A(H1N1)pdm09, A(H3N2), and influenza B virus. (PDF)

S1 Table The accession numbers of neuraminidase sequences used in this study. (PDF)

S2 Table Clinical data and sequencing results of patients with oseltamivir resistant (275Y) A(H1N1)pdm09. (PDF)

S3 Table Summary of clinical data of 18 patients with NAI-resistant seasonal influenza virus, 2009–2015. (PDF)

S4 Table Positively selected sites on the neuraminidase of A(H1N1)pdm09, seasonal A(H1N1), A(H3N2), and influenza B virus strains in Thailand, 2009–2015. (PDF)
