# Supplementary material for: Evolution of the neuraminidase gene of seasonal influenza A and B viruses in Thailand between 2010 and 2015
Source: PLoS One. 2017 Apr 14;12(4):e0175655. doi: 10.1371/journal.pone.0175655 (PMC5391933; doi:10.1371/journal.pone.0175655)
Supplement: S1 Fig — (PDF) [file pone.0175655.s001.pdf]

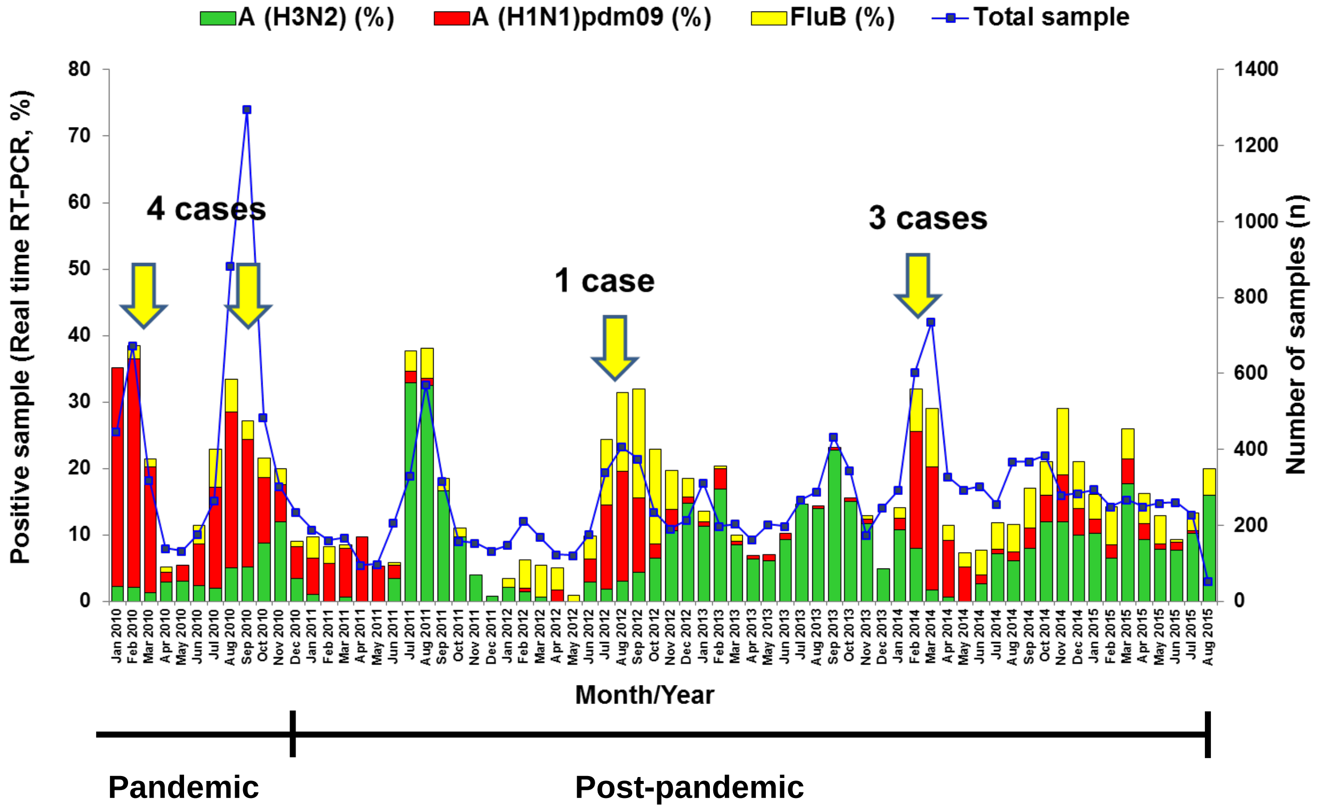

pH1N1	911 cases	485 cases
H275Y	4 (0.44%)	4 (0.82%)

S1 Fig. Incidence of influenza A and B viruses identified from clinical samples between 2010 and 2015 (N= 19,859). A total of 3,995 (20.12%) influenza viruses were isolated in Thailand. 1581 (39.57%), 1,673 (41.88%), and 741 (18.55%) were subtyped as influenza A (H3N2), A (H1N1)pdm09, and influenza B viruses, respectively. Post-pandemic of A/H1N1 pdm09, 4 of the 485 samples (0.82%) were positive for the oseltamivir resistant strains.
