# Supplementary material for: Evolution of the neuraminidase gene of seasonal influenza A and B viruses in Thailand between 2010 and 2015
Source: PLoS One. 2017 Apr 14;12(4):e0175655. doi: 10.1371/journal.pone.0175655 (PMC5391933; doi:10.1371/journal.pone.0175655)
Supplement: S2 Fig — (PDF) [file pone.0175655.s002.pdf]

S2 Fig. A maximum clade credibility tree from Bayesian timescale phylogenetic analysis of NA genes (dataset N1 seasonal n = 144 sequences, length = 1410 nt.; N1 pdm09 n = 306 sequences, length = 1407 nt.; N2 n = 373 sequences, length = 1407 nt.; NB n = 255 sequences, length = 1398 nt.). The posterior probabilities and node ages of the key nodes are depicted above the respective nodes. The major recent clusters are marked by vertical lines.

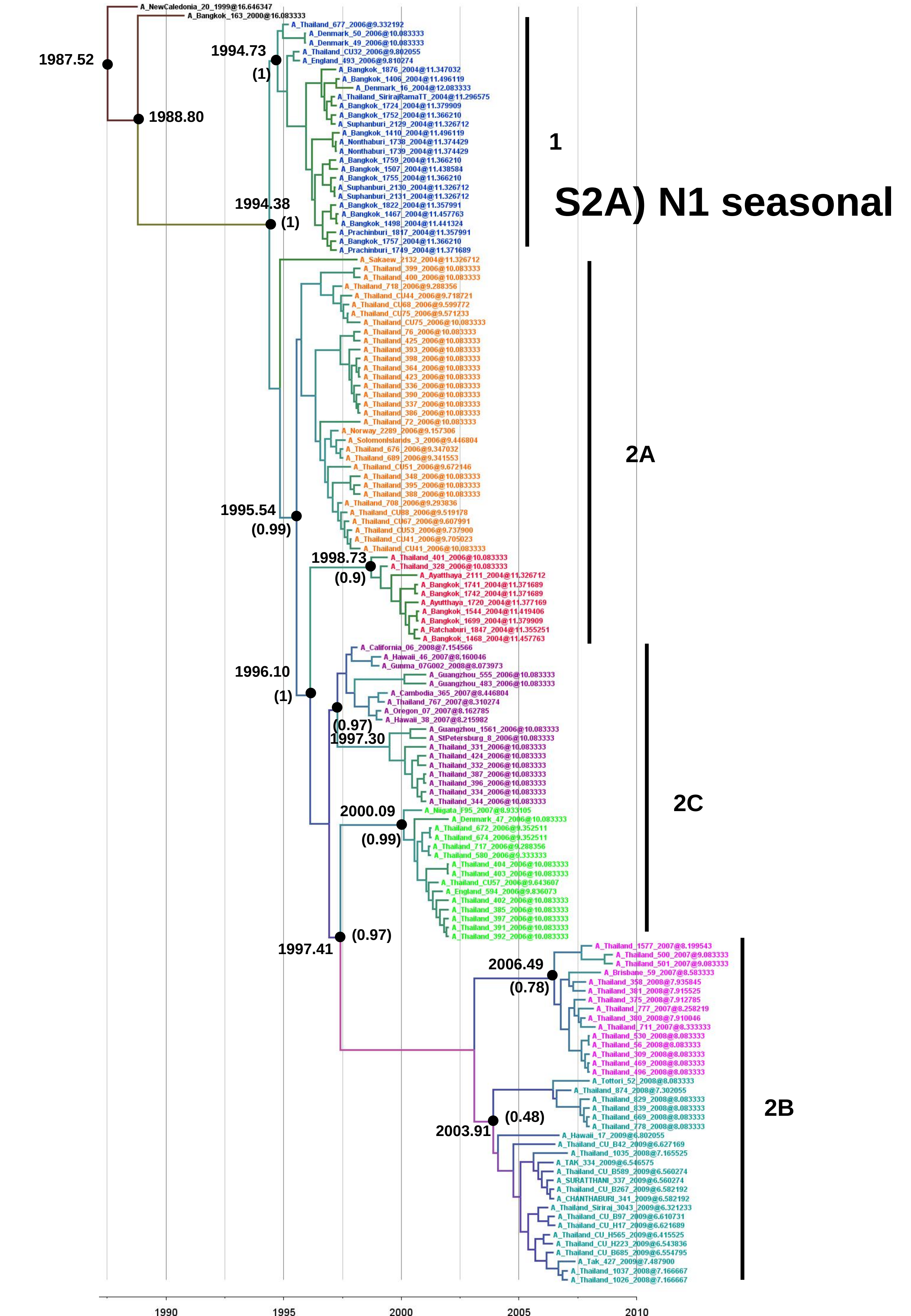
S2A) N1 seasonal
1
2A
2C
2B
1987.52
1988.80
1994.73
(1)
1994.38
(1)
1995.54
(0.99)
1998.73
(0.9)
1996.10
(1)
(0.97)
1997.30
2000.09
(0.99)
(0.97)
1997.41
2006.49
(0.78)
(0.48)
2003.91
A_NewCaledonia_20_1999@16.646347
A_Bangkok_163_2000@16.083333
A_Thailand_677_2006@9.332192
A_Denmark_50_2006@10.083333
A_Denmark_49_2006@10.083333
A_Thailand_CU32_2006@9.802055
A_England_493_2006@9.810274
A_Bangkok_1876_2004@11.347032
A_Bangkok_1406_2004@11.496119
A_Denmark_16_2004@12.083333
A_Thailand_SirirajRamaTT_2004@11.296575
A_Bangkok_1724_2004@11.379909
A_Bangkok_1752_2004@11.366210
A_Suphanburi_2129_2004@11.326712
A_Bangkok_1410_2004@11.496119
A_Nonthaburi_1738_2004@11.374429
A_Nonthaburi_1739_2004@11.374429
A_Bangkok_1759_2004@11.366210
A_Bangkok_1507_2004@11.438584
A_Bangkok_1755_2004@11.366210
A_Suphanburi_2130_2004@11.326712
A_Suphanburi_2131_2004@11.326712
A_Bangkok_1822_2004@11.357991
A_Bangkok_1467_2004@11.457763
A_Bangkok_1498_2004@11.441324
A_Prachinburi_1817_2004@11.357991
A_Bangkok_1757_2004@11.366210
A_Prachinburi_1749_2004@11.371689
A_Sakaew_2132_2004@11.326712
A_Thailand_399_2006@10.083333
A_Thailand_400_2006@10.083333
A_Thailand_718_2006@9.288356
A_Thailand_CU44_2006@9.718721
A_Thailand_CU68_2006@9.599772
A_Thailand_CU75_2006@9.571233
A_Thailand_CU75_2006@10.083333
A_Thailand_76_2006@10.083333
A_Thailand_425_2006@10.083333
A_Thailand_393_2006@10.083333
A_Thailand_398_2006@10.083333
A_Thailand_364_2006@10.083333
A_Thailand_423_2006@10.083333
A_Thailand_336_2006@10.083333
A_Thailand_390_2006@10.083333
A_Thailand_337_2006@10.083333
A_Thailand_386_2006@10.083333
A_Thailand_72_2006@10.083333
A_Norway_2289_2006@9.157306
A_SolomonIslands_3_2006@9.446804
A_Thailand_676_2006@9.347032
A_Thailand_689_2006@9.341553
A_Thailand_CU51_2006@9.672146
A_Thailand_348_2006@10.083333
A_Thailand_395_2006@10.083333
A_Thailand_388_2006@10.083333
A_Thailand_708_2006@9.293836
A_Thailand_CU88_2006@9.519178
A_Thailand_CU67_2006@9.607991
A_Thailand_CU53_2006@9.737900
A_Thailand_CU41_2006@9.705023
A_Thailand_CU41_2006@10.083333
A_Thailand_401_2006@10.083333
A_Thailand_328_2006@10.083333
A_Ayatthaya_2111_2004@11.326712
A_Bangkok_1741_2004@11.371689
A_Bangkok_1742_2004@11.371689
A_Ayutthaya_1720_2004@11.377169
A_Bangkok_1544_2004@11.419406
A_Bangkok_1699_2004@11.379909
A_Ratchaburi_1847_2004@11.355251
A_Bangkok_1468_2004@11.457763
A_California_06_2008@7.154566
A_Hawaii_46_2007@8.160046
A_Gunma_07G002_2008@8.073973
A_Guangzhou_555_2006@10.083333
A_Guangzhou_483_2006@10.083333
A_Cambodia_365_2007@8.446804
A_Thailand_767_2007@8.310274
A_Oregon_07_2007@8.162785
A_Hawaii_38_2007@8.215982
A_Guangzhou_1561_2006@10.083333
A_StPetersburg_8_2006@10.083333
A_Thailand_331_2006@10.083333
A_Thailand_424_2006@10.083333
A_Thailand_332_2006@10.083333
A_Thailand_387_2006@10.083333
A_Thailand_396_2006@10.083333
A_Thailand_334_2006@10.083333
A_Thailand_344_2006@10.083333
A_Niigata_F95_2007@8.933105
A_Denmark_47_2006@10.083333
A_Thailand_672_2006@9.352511
A_Thailand_674_2006@9.352511
A_Thailand_717_2006@9.288356
A_Thailand_580_2006@9.333333
A_Thailand_404_2006@10.083333
A_Thailand_403_2006@10.083333
A_Thailand_CU57_2006@9.643607
A_England_594_2006@9.836073
A_Thailand_402_2006@10.083333
A_Thailand_385_2006@10.083333
A_Thailand_397_2006@10.083333
A_Thailand_391_2006@10.083333
A_Thailand_392_2006@10.083333
A_Thailand_1577_2007@8.199543
A_Thailand_500_2007@9.083333
A_Thailand_501_2007@9.083333
A_Brisbane_59_2007@8.583333
A_Thailand_358_2008@7.935845
A_Thailand_381_2008@7.915525
A_Thailand_375_2008@7.912785
A_Thailand_777_2007@8.258219
A_Thailand_380_2008@7.910046
A_Thailand_711_2007@8.333333
A_Thailand_530_2008@8.083333
A_Thailand_56_2008@8.083333
A_Thailand_309_2008@8.083333
A_Thailand_469_2008@8.083333
A_Thailand_496_2008@8.083333
A_Tottori_52_2008@8.083333
A_Thailand_874_2008@7.302055
A_Thailand_829_2008@8.083333
A_Thailand_839_2008@8.083333
A_Thailand_669_2008@8.083333
A_Thailand_778_2008@8.083333
A_Hawaii_17_2009@6.802055
A_Thailand_CU_B42_2009@6.627169
A_Thailand_1035_2008@7.165525
A_TAK_334_2009@6.546575
A_Thailand_CU_B589_2009@6.560274
A_SURATTHANI_337_2009@6.560274
A_Thailand_CU_B267_2009@6.582192
A_CHANTHABURI_341_2009@6.582192
A_Thailand_Siriraj_3043_2009@6.321233
A_Thailand_CU_B97_2009@6.610731
A_Thailand_CU_H17_2009@6.621689
A_Thailand_CU_H565_2009@6.415525
A_Thailand_CU_H223_2009@6.543836
A_Thailand_CU_B685_2009@6.554795
A_Tak_427_2009@7.487900
A_Thailand_1037_2008@7.166667
A_Thailand_1026_2008@7.166667
1990
1995
2000
2005
2010

S2B) N1 pdm09

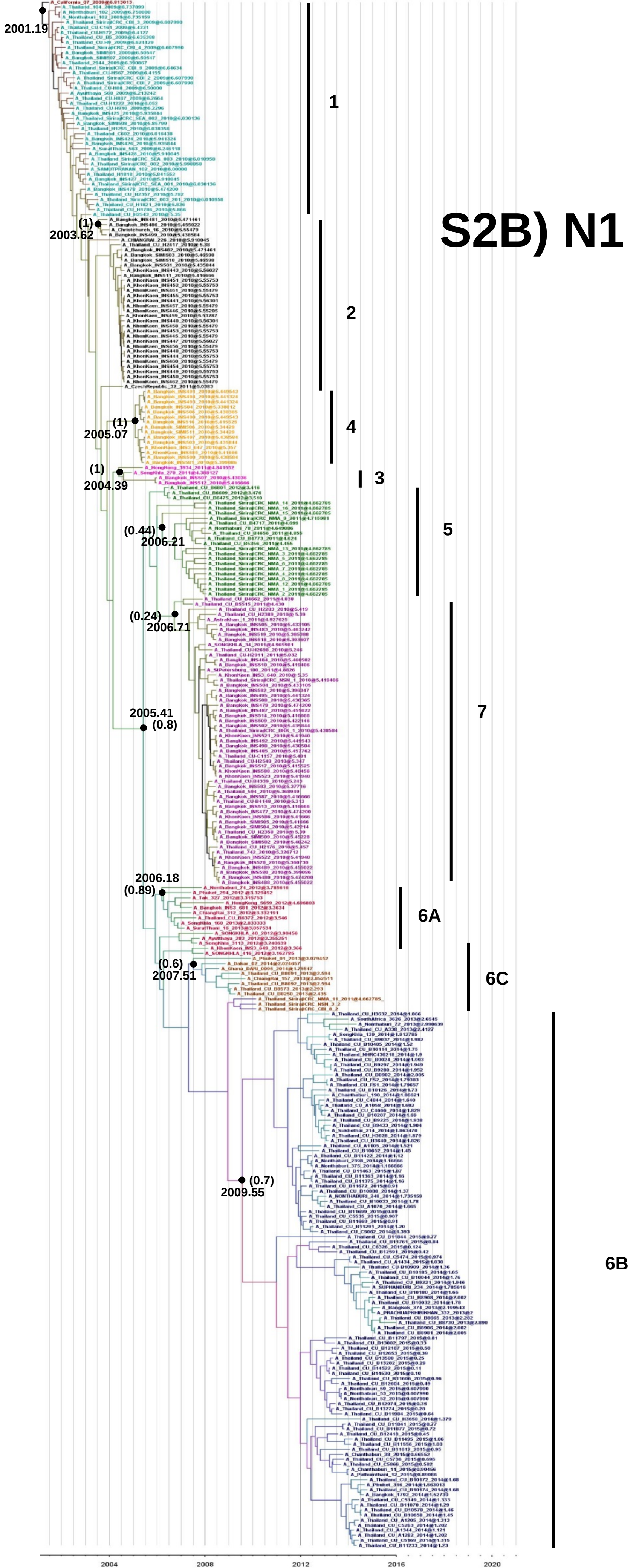
2001.19
(1)
2003.62
(1)
2005.07
(1)
2004.39
(0.44)
2006.21
(0.24)
2006.71
2005.41
(0.8)
2006.18
(0.89)
(0.6)
2007.51
(0.7)
2009.55
1
2
4
3
5
7
6A
6C
6B
2004
2008
2012
2016
2020

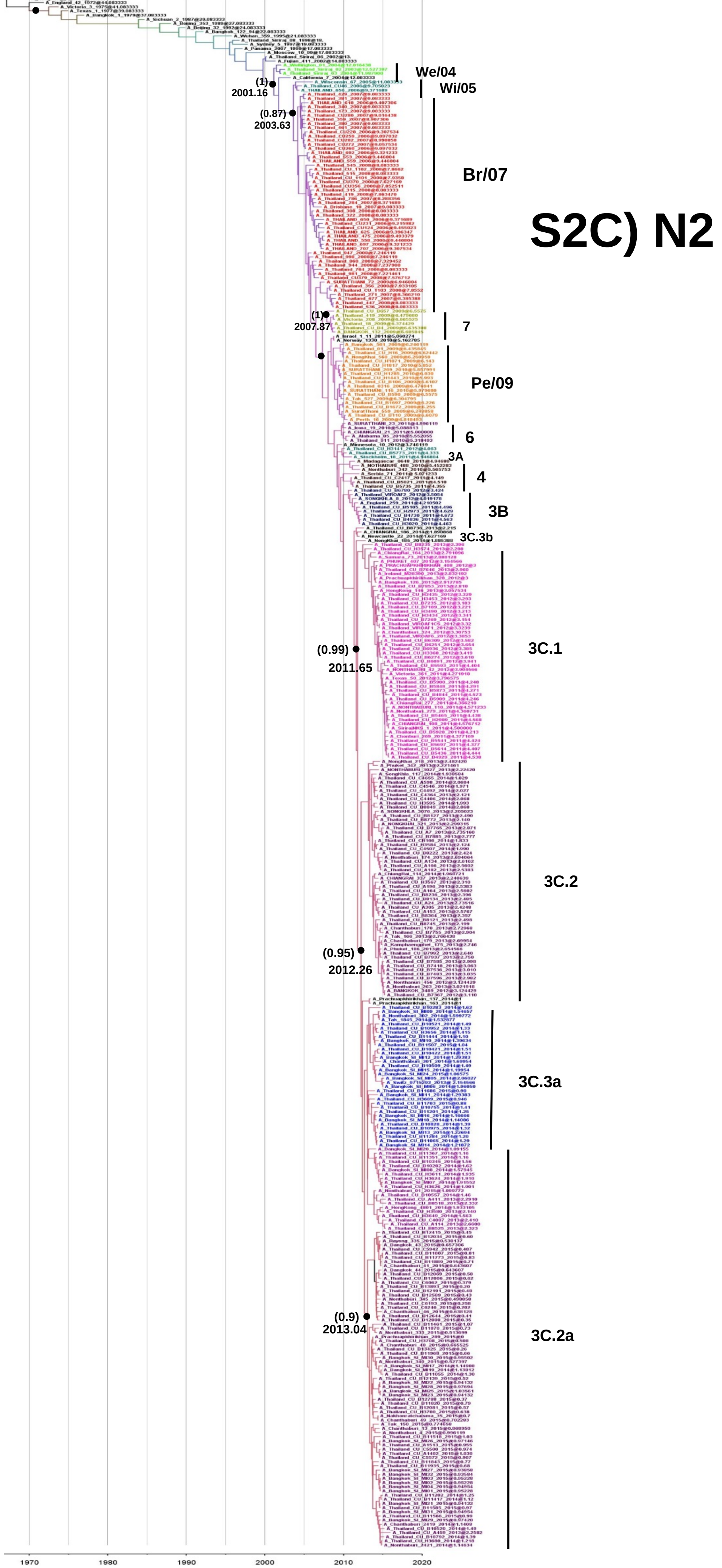
S2C) N2
We/04
Wi/05
Br/07
7
Pe/09
6
3A
4
3B
3C.3b
3C.1
3C.2
3C.3a
3C.2a
(1)
2001.16
(0.87)
2003.63
(1)
2007.87
(0.99)
2011.65
(0.95)
2012.26
(0.9)
2013.04
1970
1980
1990
2000
2010
2020

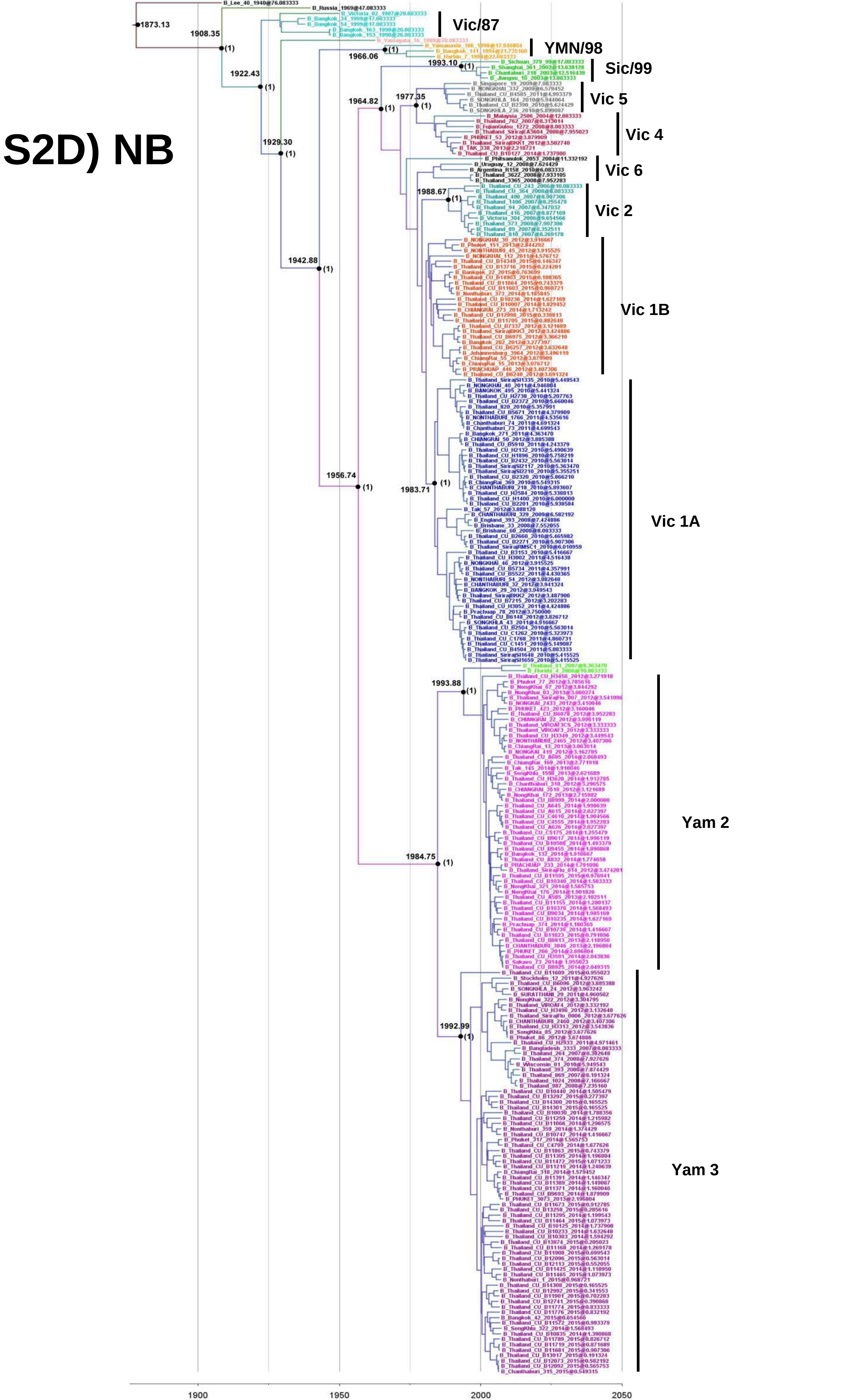
S2D) NB
Vic/87
YMN/98
Sic/99
Vic 5
Vic 4
Vic 6
Vic 2
Vic 1B
Vic 1A
Yam 2
Yam 3
1873.13
1908.35
1922.43
1929.30
1942.88
1956.74
1964.82
1966.06
1977.35
1983.71
1984.75
1988.67
1992.99
1993.10
1993.88
1900
1950
2000
2050
