# Supplementary material for: Evolution of the neuraminidase gene of seasonal influenza A and B viruses in Thailand between 2010 and 2015
Source: PLoS One. 2017 Apr 14;12(4):e0175655. doi: 10.1371/journal.pone.0175655 (PMC5391933; doi:10.1371/journal.pone.0175655)
Supplement: S3 Fig — (PDF) [file pone.0175655.s003.pdf]

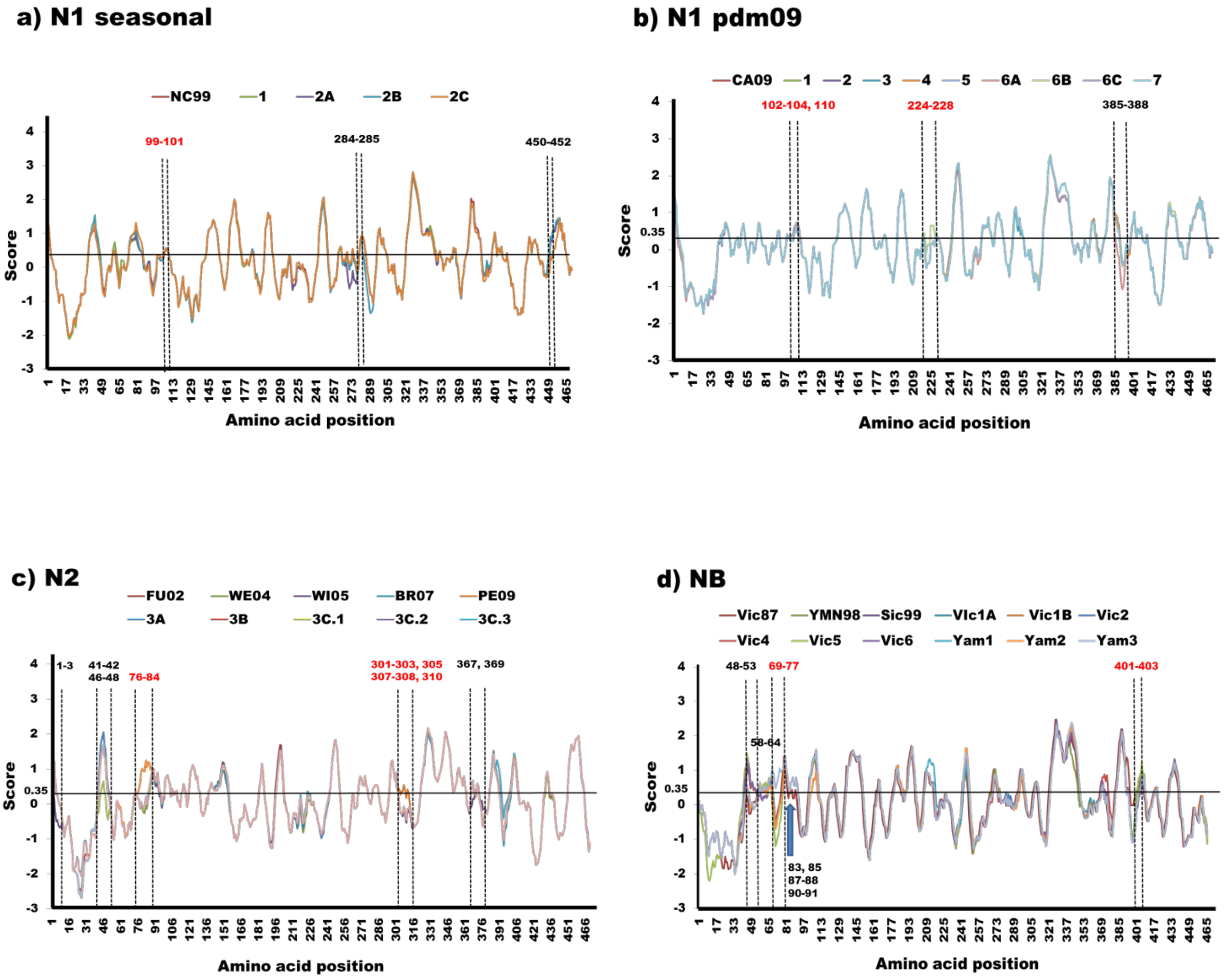

S3 Fig. Predicted B-cell epitopes of NA proteins of influenza A/H1N1 seasonal (a), A/H1N1 pdm09 (b), A/H3N2 (c), and influenza B (d) viruses. The BepiPred score above the 0.35 threshold is shown by horizontal line. Epitope difference between the vaccine (A/New Caledonia/20/1999 for N1 seasonal, A/California/07/2009 for N1 pdm09, A/Fujian/411/2002 for N2, and B/Victoria/02/1987 for NB) and Thai strains are indicated by dotted vertical lines. The substituted amino acid contribute to loss B-cell epitope are denoted a black letter, while that gain of the antigen property are denoted red letter.
