# Supplementary material for: Evolution of the neuraminidase gene of seasonal influenza A and B viruses in Thailand between 2010 and 2015
Source: PLoS One. 2017 Apr 14;12(4):e0175655. doi: 10.1371/journal.pone.0175655 (PMC5391933; doi:10.1371/journal.pone.0175655)
Supplement: S1 Table — (PDF) [file pone.0175655.s004.pdf]

S1 Table. The accession numbers of neuraminidase sequences were used in present study

Influenza A(H3N2)

Virus name	Accession number
A/California/7/2004	EPI367106
A/Wisconsin/67/2005	EPI160220
A/Brisbane/10/2007	EPI165491
A/Perth/16/2009	EPI182942
A/Victoria/361/2011	EPI353905
A/Switzerland/9715293/2013	EPI540525
A/Moscow/10/99	EPI103336
A/Panama/2007/1999	DQ487337
A/Beijing/353/1989	DQ508835
A/Beijing/32/1992	CY033608
A/England/42/1972	CY113087
A/Fujian/411/2002	CY088485
A/Hong Kong/1/1968	CY112251
A/Sichuan/2/1987	CY112398
A/Sydney/5/1997	CY112887
A/Texas/1/1977	CY113263
A/Victoria/3/1975	CY113183
A/Wuhan/359/1995	CY112823
A/Wellington/01/2004	CY012106
A/Israel/1/11/2011	EPI319251
A/Norway/1330/2010	EPI302230
A/Stockholm/18/2011	EPI318273
A/England/259/2011	EPI346608
A/Texas/50/2012	KC892237
A/Hong Kong/146/2013	EPI426062
A/Ireland/M28390/2013	EPI467995
A/Hong Kong/4801/2014	EPI539577
A/Samara/73/2013	EPI460559
A/Newcastle/22/2014	EPI541460
A/Madagascar/0648/2011	EPI319277
A/Serbia/71/2011	EPI326116
A/Alabama/05/2010	KC535442
A/Iowa/19/2010	KC882859
A/Minnesota/10/2012	KC892316
A/Victoria/208/2009	EPI232452
A/Bangkok/1/1979 1979	CY121002

A/Bangkok/SI-MI01/2015	KP877373
A/Bangkok/SI-MI02/2015	KP877374
A/Bangkok/SI-MI03/2015	KP877375
A/Bangkok/SI-MI04/2015	KP877376
A/Bangkok/SI-MI05/2014	KP877377
A/Bangkok/SI-MI06/2014	KP877378
A/Bangkok/SI-MI07/2014	KP877379
A/Bangkok/SI-MI08/2014	KP877380
A/Bangkok/SI-MI09/2014	KP877381
A/Bangkok/SI-MI10/2014	KP877382
A/Bangkok/SI-MI11/2014	KP877383
A/Bangkok/SI-MI12/2014	KP877384
A/Bangkok/SI-MI13/2014	KP877385
A/Bangkok/SI-MI14/2014	KP877386
A/Bangkok/SI-MI15/2014	KP877387
A/Bangkok/SI-MI16/2014	KP877388
A/Bangkok/SI-MI17/2014	KP877389
A/Bangkok/SI-MI18/2014	KP877390
A/Bangkok/SI-MI19/2014	KP877391
A/Bangkok/SI-MI20/2014	KP877392
A/Bangkok/SI-MI21/2015	KP877393
A/Bangkok/SI-MI22/2015	KP877394
A/Bangkok/SI-MI23/2015	KP877395
A/Bangkok/SI-MI24/2015	KP877396
A/Bangkok/SI-MI25/2015	KP877397
A/Bangkok/SI-MI26/2015	KP877398
A/Bangkok/SI-MI27/2015	KP877399
A/Bangkok/SI-MI28/2015	KP877400
A/Bangkok/SI-MI29/2015	KP877401
A/Bangkok/SI-MI30/2015	KP877402
A/Bangkok/SI-MI31/2015	KP877403
A/Bangkok/SI-MI32/2015	KP877404
A/Thailand/173/2007	AB501496
A/Thailand/308/2008	AB501490
A/Thailand/315/2008	AB501497
A/Thailand/322/2008	AB501493
A/Thailand/340/2007	AB501499
A/Thailand/380/2007	AB501503
A/Thailand/381/2007	AB501504
A/Thailand/429/2007	AB501506
A/Thailand/447/2008	AB501491

A/Thailand/461/2007	AB501507
A/Thailand/515/2008	AB501487
A/Thailand/536/2008	AB501498
A/Thailand/545/2008	AB501488
A/Thailand/764/2008	AB501508
A/Thailand/CU-1101/2008	EU625366
A/Thailand/CU-1102/2008	EU625367
A/Thailand/CU-1103/2008	EU625368
A/Thailand/CU-A114/2013	KP336092
A/Thailand/CU-A134/2013	KP336094
A/Thailand/CU-A153/2013	KP336095
A/Thailand/CU-A164/2013	KP336097
A/Thailand/CU-A166/2013	KP336096
A/Thailand/CU-A182/2013	KP336099
A/Thailand/CU-A196/2013	KP336098
A/Thailand/CU-A24/2013	KP336091
A/Thailand/CU-A305/2013	KP336103
A/Thailand/CU-A411/2013	KP336112
A/Thailand/CU-A459/2013	KP336114
A/Thailand/CU-A598/2014	KP336121
A/Thailand/CU-A7/2013	KP336090
A/Thailand/CU-B10282/2014	KP336134
A/Thailand/CU-B10283/2014	KP336133
A/Thailand/CU-B10345/2014	KP336136
A/Thailand/CU-B10421/2014	KP336137
A/Thailand/CU-B10422/2014	KP336138
A/Thailand/CU-B10509/2014	KP336139
A/Thailand/CU-B10520/2014	KP336141
A/Thailand/CU-B10521/2014	KP336140
A/Thailand/CU-B10557/2014	KP336142
A/Thailand/CU-B106/2009	GQ983550
A/Thailand/CU-B10755/2014	KP336144
A/Thailand/CU-B10792/2014	KP336145
A/Thailand/CU-B10828/2014	KP336146
A/Thailand/CU-B10952/2014	KP336147
A/Thailand/CU-B10975/2014	KP336148
A/Thailand/CU-B110/2009	GQ902811
A/Thailand/CU-B11055/2014	KP336149
A/Thailand/CU-B11065/2014	KP336150
A/Thailand/CU-B11201/2014	KP336151
A/Thailand/CU-B11202/2014	KP336152

A/Thailand/CU-B11284/2014	KP336154
A/Thailand/CU-B11351/2014	KP336155
A/Thailand/CU-B11367/2014	KP336156
A/Thailand/CU-B1672/2009	GU271976
A/Thailand/CU-B1697/2009	GU271984
A/Thailand/CU-B4/2009	GQ902795
A/Thailand/CU-B4730/2011	KP336040
A/Thailand/CU-B4836/2011	KP336044
A/Thailand/CU-B4844/2011	KP336042
A/Thailand/CU-B4929/2011	KP336045
A/Thailand/CU-B5021/2011	KP336046
A/Thailand/CU-B5105/2011	KP336047
A/Thailand/CU-B5436/2011	KP336049
A/Thailand/CU-B5465/2011	KP336050
A/Thailand/CU-B5541/2011	KP336051
A/Thailand/CU-B5593/2011	KP336053
A/Thailand/CU-B5614/2011	KP336052
A/Thailand/CU-B5697/2011	KP336054
A/Thailand/CU-B5735/2011	KP336055
A/Thailand/CU-B5773/2011	KP336056
A/Thailand/CU-B5848/2011	KP336057
A/Thailand/CU-B5873/2011	KP336058
A/Thailand/CU-B590/2009	GQ902819
A/Thailand/CU-B5900/2011	KP336059
A/Thailand/CU-B5909/2011	KP336060
A/Thailand/CU-B5928/2011	KP336061
A/Thailand/CU-B6091/2012	KP336064
A/Thailand/CU-B6251/2012	KP336065
A/Thailand/CU-B6274/2012	KP336066
A/Thailand/CU-B6309/2012	KP336067
A/Thailand/CU-B657/2009	GQ902827
A/Thailand/CU-B6780/2012	KP336068
A/Thailand/CU-B6936/2012	KP336070
A/Thailand/CU-B7189/2012	KP336074
A/Thailand/CU-B7235/2012	KP336076
A/Thailand/CU-B7269/2012	KP336077
A/Thailand/CU-B7367/2012	KP336078
A/Thailand/CU-B7418/2013	KP336079
A/Thailand/CU-B7483/2013	KP336080
A/Thailand/CU-B7536/2013	KP336081
A/Thailand/CU-B7585/2013	KP336082

A/Thailand/CU-B7596/2013	KP336083
A/Thailand/CU-B7646/2013	KP336084
A/Thailand/CU-B7755/2013	KP336085
A/Thailand/CU-B7765/2013	KP336086
A/Thailand/CU-B7853/2013	KP336087
A/Thailand/CU-B7885/2013	KP336088
A/Thailand/CU-B7937/2013	KP336089
A/Thailand/CU-B7992/2013	KP336093
A/Thailand/CU-B8121/2013	KP336100
A/Thailand/CU-B8127/2013	KP336101
A/Thailand/CU-B8134/2013	KP336102
A/Thailand/CU-B8222/2013	KP336104
A/Thailand/CU-B8235/2013	KP336106
A/Thailand/CU-B8236/2013	KP336107
A/Thailand/CU-B8364/2013	KP336108
A/Thailand/CU-B8518/2013	KP336109
A/Thailand/CU-B8525/2013	KP336110
A/Thailand/CU-B8736/2013	KP336115
A/Thailand/CU-B8745/2013	KP336116
A/Thailand/CU-B8772/2013	KP336118
A/Thailand/CU-B8849/2014	KP336122
A/Thailand/CU-C2417/2011	KP336062
A/Thailand/CU-C4087/2013	KP336105
A/Thailand/CU-C4364/2013	KP336120
A/Thailand/CU-C4406/2014	KP336123
A/Thailand/CU-C4492/2014	KP336124
A/Thailand/CU-C4507/2014	KP336126
A/Thailand/CU-C4546/2014	KP336127
A/Thailand/CU-C4655/2014	KP336132
A/Thailand/CU-CB166/2014	KP336131
A/Thailand/CU-H1071/2009	CY074944
A/Thailand/CU-H1285/2010	CY074952
A/Thailand/CU-H1443/2010	CY074960
A/Thailand/CU-H16/2009	GU271992
A/Thailand/CU-H1817/2010	CY074968
A/Thailand/CU-H2973/2011	KP336041
A/Thailand/CU-H2989/2011	KP336043
A/Thailand/CU-H3020/2011	KP336048
A/Thailand/CU-H3141/2012	KP336063
A/Thailand/CU-H3368/2012	KP336069
A/Thailand/CU-H3434/2012	KP336071

A/Thailand/CU-H3435/2012	KP336072
A/Thailand/CU-H3453/2012	KP336073
A/Thailand/CU-H3490/2012	KP336075
A/Thailand/CU-H3567/2013	KP336111
A/Thailand/CU-H3574/2013	KP336113
A/Thailand/CU-H3580/2013	KP336117
A/Thailand/CU-H3584/2013	KP336119
A/Thailand/CU-H3595/2014	KP336125
A/Thailand/CU-H3611/2014	KP336128
A/Thailand/CU-H3624/2014	KP336129
A/Thailand/CU-H3626/2014	KP336130
A/Thailand/CU-H3649/2014	KP336135
A/Thailand/CU-H3656/2014	KP336143
A/Thailand/CU-H3680/2014	KP336153
A/Thailand/CU-124/2006	EU021285
A/Thailand/CU-228/2006	EU021275
A/Thailand/CU-231/2006	EU021283
A/Thailand/CU-259/2006	EU021279
A/Thailand/CU-260/2006	EU021281
A/Thailand/CU-272/2007	EU021271
A/Thailand/CU-280/2007	EU021273
A/Thailand/CU-282/2007	EU021277
A/Thailand/CU-356/2008	FJ912978
A/Thailand/CU-370/2008	FJ912986
A/Thailand/CU-379/2008	FJ912994
A/Thailand/CU-46/2006	EU021269
A/Thailand/Siriraj/02/2003	JN617983
A/Thailand/Siriraj/03/2004	JN617986
A/Thailand/Siriraj/06/2002	JN617984
A/Thailand/Siriraj/08/1998	JN617985
A/Thailand/VIROAF1/2012	KJ577151
A/Thailand/VIROAF1CS/2012	KJ848676
A/Thailand/VIROAF2/2012	KJ577159
A/Thailand/VIROAF6/2012	KJ577191
A/Bangkok/122/94	U43426
A/Siriraj/ICRC/NKS/1/2011	EPI670324
A/Tak/150/2015	EPI652271
A/Chanthaburi/13/2015	EPI652262
A/Nonthaburi/4/2015	EPI652256
A/Chanthaburi/49/2015	EPI649730
A/Bangkok/44/2015	EPI649660

A/Chanthaburi/41/2015	EPI649642
A/Bangkok/43/2015	EPI649619
A/Chanthaburi/40/2015	EPI649556
A/Chanthaburi/46/2015	EPI649548
A/Nakhonratchaisma/35/2015	EPI649505
A/Prachuapkhirikhan/289/2015	EPI649489
A/Nonthaburi/345/2015	EPI647891
A/Nonthaburi/340/2015	EPI647883
A/Rayong/335/2015	EPI647875
A/Nonthaburi/333/2015	EPI647859
A/Nong Khai/218/2013	EPI587902
A/Phuket/186/2013	EPI587901
A/Chanthaburi/179/2013	EPI587900
A/Tak/166/2013	EPI587899
A/Chiang Rai/164/2013	EPI587897
A/Bangkok/126/2013	EPI587896
A/NongKhai/185/2014	EPI567266
A/Chanthaburi/2419/2014	EPI566016
A/Nonthaburi/2421/2014	EPI565992
A/Nonthaburi/01/2015	EPI564286
A/Chanthaburi/170/2013	EPI553214
A/Phuket/342/2013	EPI553203
A/Tak/1845/2014	EPI545580
A/Nonthaburi/302/2014	EPI543716
A/Chanthaburi/301/2014	EPI543707
A/CHIANGRAI/186/2014	EPI541336
A/SONGKHLA/3076/2013	EPI529586
A/NONTHABURI/3027/2013	EPI529570
A/CHIANGRAI/337/2013	EPI529567
A/NONGKHAI/321/2013	EPI529559
A/BANGKOK/3489/2012	EPI526660
A/Prachuapkhirikhan/137/2014	EPI520396
A/Prachuapkhirikhan/163/2014	EPI520358
A/SongKhla/117/2014	EPI516707
A/ChiangRai/114/2014	EPI516704
A/Phuket/186/2013	EPI467241
A/Kamphaengphet/175/2013	EPI466932
A/Nonthaburi/174/2013	EPI466923
A/PRACHUAPKHIRIKHAN/408/2012	EPI450270
A/PHUKET/407/2012	EPI450267
A/Nonthanuri/456/2012	EPI432651

A/Nonthaburi/263/2013	EPI432622
A/Prachuapkhirikhan/328/2012	EPI406056
A/Chanthaburi/324/2012	EPI406047
A/NONTHABURI/42/2012	EPI379390
A/SONGKHLA/8/2012	EPI379384
A/ChiangRai/277/2011	EPI348477
A/Nonthaburi/279/2011	EPI346457
A/ChiangRai/277/2011	EPI346454
A/Chonburi/269/2011	EPI346451
A/NONTHABURI/110/2011	EPI346319
A/CHIANGRAI/108/2011	EPI346316
A/CHIANGRAI/21/2011	EPI331593
A/SURATTHANI/23/2011	EPI331542
A/Thailand/911/2010	EPI295270
A/NOTHABURI/488/2010	EPI294227
A/Nonthaburi/342/2010	EPI278795
A/SURATTHANI/269/2010	EPI272024
A/SURATTHANI/116/2010	EPI272018
A/Bangkok/561/2009	EPI232664
A/Thailand/419/2009	EPI232622
A/Thailand/18/2009	EPI232619
A/Thailand/0316/2009	EPI232616
A/Thailand/01/2009	EPI232613
A/Tak/527/2009	EPI232607
A/SuratThani/559/2009	EPI232601
A/NongKhai/560/2009	EPI232584
A/BANGKOK/132/2009	EPI228245
A/SURATTHANI/72/2009	EPI211733
A/Thailand/981/2008	EPI185822
A/Thailand/947/2008	EPI185821
A/Thailand/860/2008	EPI185819
A/Thailand/998/2008	EPI175304
A/Thailand/944/2008	EPI175299
A/Thailand/419/2008	EPI163138
A/Thailand/356/2008	EPI163136
A/Thailand/786/2007	EPI162314
A/Thailand/677/2007	EPI162302
A/Thailand/284/2007	EPI162299
A/Thailand/271/2007	EPI162296
A/Thailand/786/2007	EPI158244
A/Thailand/677/2007	EPI158232

A/Thailand/284/2007	EPI158223
A/Thailand/271/2007	EPI158220
A/Thailand/359/2007	EPI155714
A/THAILAND/656/2006	EPI155273
A/THAILAND/650/2006	EPI155271
A/THAILAND/707/2006	EPI155260
A/THAILAND/697/2006	EPI155256
A/THAILAND/692/2006	EPI155254
A/THAILAND/625/2006	EPI155250
A/THAILAND/618/2006	EPI155248
A/THAILAND/559/2006	EPI155246
A/Thailand/553/2006	EPI155244
A/THAILAND/558/2006	EPI155242
A/THAILAND/475/2006	EPI155240
A/Thailand/CU-B11417/2014	KX151186
A/Thailand/CU-B11444/2014	KX151187
A/Thailand/CU-B11461/2015	KX151188
A/Thailand/CU-B11507/2015	KX151189
A/Thailand/CU-B11518/2915	KX151190
A/Thailand/CU-A1402/2015	KX151191
A/Thailand/CU-B11566/2015	KX151192
A/Thailand/CU-B11585/2015	KX151193
A/Thailand/CU-C5500/2015	KX151194
A/Thailand/CU-A1513/2015	KX151195
A/Thailand/CU-H3689/2015	KX151196
A/Thailand/CU-C5572/2015	KX151197
A/Thailand/CU-B11686/2015	KX151198
A/Thailand/CU-B11703/2015	KX151199
A/Thailand/CU-B11773/2015	KX151200
A/Thailand/CU-B11807/2015	KX151201
A/Thailand/CU-B11820/2015	KX151202
A/Thailand/CU-B11843/2015	KX151203
A/Thailand/CU-B11870/2015	KX151204
A/Thailand/CU-B11889/2015	KX151205
A/Thailand/CU-B11935/2015	KX151206
A/Thailand/CU-B11968/2015	KX151207
A/Thailand/CU-H3700/2015	KX151208
A/Thailand/CU-B12006/2015	KX151209
A/Thailand/CU-B12034/2015	KX151210
A/Thailand/CU-B12069/2015	KX151211
A/Thailand/CU-B12081/2015	KX151212

A/Thailand/CU-B12139/2015	KX151213
A/Thailand/CU-H3708/2015	KX151214
A/Thailand/CU-C5942/2015	KX151215
A/Thailand/CU-B12191/2015	KX151216
A/Thailand/CU-B12415/2015	KX151217
A/Thailand/CU-B12589/2015	KX151218
A/Thailand/CU-B12644/2015	KX151219
A/Thailand/CU-C6062/2015	KX151220
A/Thailand/CU-B12788/2015	KX151221
A/Thailand/CU-B12888/2015	KX151222
A/Thailand/CU-B13425/2015	KX151223
A/Thailand/CU-C6193/2015	KX151224
A/Thailand/CU-C6246/2015	KX151225
A/Thailand/CU-B13893/2015	KX151226

Influenza A(H1N1)

Virus name	Accession number
A/NewCaledonia/20/1999	CY033624
A/Denmark/16/2004	EU097726
A/England/493/2006	FJ445052
A/Denmark/49/2006	EU097736
A/Denmark/50/2006	EU097737
A/Norway/2289/2006	EPI509388
A/Thailand/CU41/2006	EU021247
A/Solomon Islands/3/2006	EPI509400
A/StPetersburg/8/2006	CY035128
A/Thailand/CU75/2006	EU021263
A/Guangzhou/1561/2006	EU382993
A/Guangzhou/483/2006	EU382988
A/Guangzhou/555/2006	EU382990
A/Cambodia/365/2007	EPI157505
A/Hawaii/38/2007	EU516270
A/Oregon/07/2007	EU516285
A/Hawaii/46/2007	EU779656
A/California/06/2008	GQ475837
A/Gunma/07G002/2008	CY043411
A/Denmark/47/2006	EU097731
A/England/594/2006	FJ445066
A/Niigata/F95/2007	CY094798

A/Brisbane/59/2007	CY058489
A/Tottori/52/2008	CY075491
A/Hawaii/17/2009	EPI189495
A/Bangkok/163/2000	CY125110
A/Thailand/1035/2008	GQ423407
A/Thailand/309/2008	AB501444
A/Thailand/328/2006	AB501445
A/Thailand/331/2006	AB501446
A/Thailand/332/2006	AB501448
A/Thailand/332/2008	AB501447
A/Thailand/334/2006	AB501449
A/Thailand/336/2006	AB501450
A/Thailand/337/2006	AB501451
A/Thailand/344/2006	AB501452
A/Thailand/348/2006	AB501453
A/Thailand/364/2006	AB501454
A/Thailand/385/2006	AB501455
A/Thailand/386/2006	AB501456
A/Thailand/387/2006	AB501457
A/Thailand/388/2006	AB501458
A/Thailand/390/2006	AB501459
A/Thailand/391/2006	AB501460
A/Thailand/392/2006	AB501461
A/Thailand/393/2006	AB501462
A/Thailand/395/2006	AB501463
A/Thailand/396/2006	AB501464
A/Thailand/397/2006	AB501465
A/Thailand/398/2006	AB501466
A/Thailand/399/2006	AB501467
A/Thailand/400/2006	AB501468
A/Thailand/401/2006	AB501469
A/Thailand/402/2006	AB501470
A/Thailand/403/2006	AB501471
A/Thailand/404/2006	AB501472
A/Thailand/423/2006	AB501473
A/Thailand/424/2006	AB501474
A/Thailand/425/2006	AB501475
A/Thailand/469/2008	AB501476
A/Thailand/496/2008	AB501477
A/Thailand/500/2007	AB501478
A/Thailand/501/2007	AB501479

A/Thailand/530/2008	AB501480
A/Thailand/56/2008	AB501481
A/Thailand/669/2008	AB526260
A/Thailand/708/2006	EU879083
A/Thailand/72/2006	AB501482
A/Thailand/76/2006	AB501483
A/Thailand/778/2008	AB501484
A/Thailand/829/2008	AB501485
A/Thailand/839/2008	AB501486
A/Thailand/CU32/2006	EU021265
A/Thailand/CU41/2006	EU021247
A/Thailand/CU44/2006	EU021259
A/Thailand/CU51/2006	EU021255
A/Thailand/CU53/2006	EU021249
A/Thailand/CU57/2006	EU021257
A/Thailand/CU67/2006	EU021251
A/Thailand/CU68/2006	EU021261
A/Thailand/CU75/2006	EU021263
A/Thailand/CU88/2006	EU021253
A/Thailand/SirirajRamaTT/2004	JN676133
A/Thailand/CU-H223/2009	GU183817
A/Thailand/CU-H565/2009	GU271968
A/Thailand/Siriraj/3043/2009	JN676131
A/Thailand/CU-B589/2009	GU183801
A/Thailand/CU-B685/2009	GU183809
A/Thailand/CU-B97/2009	GU271952
A/Thailand/CU-H17/2009	GQ902835
A/Thailand/CU-B267/2009	GU271960
A/Thailand/CU-B42/2009	GQ902803
A/Thailand/874/2008	EPI232891
A/Thailand/1026/2008	EPI232890
A/Tak/427/2009	EPI232888
A/CHANTHABURI/341/2009	EPI228273
A/SURAT THANI/337/2009	EPI228257
A/TAK/334/2009	EPI 228255
A/Thailand/1037/2008	EPI189736
A/Thailand/381/2008	EPI163216
A/Thailand/380/2008	EPI163061
A/Thailand/375/2008	EPI163059
A/Thailand/358/2008	EPI163057
A/Thailand/777/2007	EPI158238

A/Thailand/767/2007	EPI158236
A/Thailand/711/2007	EPI158234
A/Thailand/1577/2007	EPI158217
A/Suphanburi/2131/2004	EPI158162
A/Suphanburi/2130/2004	EPI158161
A/Suphanburi/2129/2004	EPI158160
A/Sakaew/2132/2004	EPI158099
A/Ratchaburi/1847/2004	EPI158089
A/Prachinburi/1817/2004	EPI158082
A/Prachinburi/1749/2004	EPI158080
A/Nonthaburi/1739/2004	EPI158031
A/Nonthaburi/1738/2004	EPI158029
A/Bangkok/1876/2004	EPI157428
A/Bangkok/1822/2004	EPI157427
A/Bangkok/1759/2004	EPI157426
A/Bangkok/1757/2004	EPI157425
A/Bangkok/1755/2004	EPI157424
A/Bangkok/1752/2004	EPI157423
A/Bangkok/1742/2004	EPI157422
A/Bangkok/1741/2004	EPI157421
A/Bangkok/1724/2004	EPI157420
A/Bangkok/1699/2004	EPI157419
A/Bangkok/1544/2004	EPI157418
A/Bangkok/1507/2004	EPI157416
A/Bangkok/1498/2004	EPI157415
A/Bangkok/1468/2004	EPI157414
A/Bangkok/1467/2004	EPI157413
A/Bangkok/1410/2004	EPI157412
A/Bangkok/1406/2004	EPI157411
A/Ayutthaya/1720/2004	EPI157410
A/Ayatthaya/2111/2004	EPI157408
A/Thailand/689/2006	EPI155285
A/Thailand/677/2006	EPI155283
A/Thailand/580/2006	EPI155281
A/Thailand/676/2006	EPI155279
A/Thailand/672/2006	EPI155277
A/Thailand/674/2006	EPI155275
A/Thailand/717/2006	EPI155268
A/Thailand/718/2006	EPI155266

Influenza A(H1N1)pdm09

Virus name	Accession number
A/California/07/2009	FJ984386
A/CzechRepublic/32/2011	EPI319448
A/HongKong/3934/2011	EPI326207
A/Christchurch/16/2010	EPI280343
A/Astrakhan/1/2011	EPI319591
A/HongKong/5659/2012	EPI382425
A/SouthAfrica/3626/2013	EPI466627
A/Dakar/02/2014	EPI539471
A/Ghana/DARI/0095/2014	EPI541030
A/StPetersburg/100/2011	EPI316434
A/Thailand/CU-B4656/2011	KX151227
A/Thailand/CU-B4662/2011	KX151228
A/Thailand/CU-B4717/2011	KX151229
A/Thailand/CU-B4773/2011	KX151230
A/Thailand/CU-B5356/2011	KX151231
A/Thailand/CU-B5515/2011	KX151232
A/Thailand/CU-B6475/2012	KX151233
A/Thailand/CU-B6609/2012	KX151234
A/Thailand/CU-B6801/2012	KX151235
A/Thailand/CU-B8091/2013	KX151236
A/Thailand/CU-B8092/2013	KX151237
A/Thailand/CU-B8250/2013	KX151238
A/Thailand/CU-A338/2013	KX151239
A/Thailand/CU-B8573/2013	KX151240
A/Thailand/CU-B8665/2013	KX151241
A/Thailand/CU-B8730/2013	KX151242
A/Thailand/CU-B8906/2014	KX151243
A/Thailand/CU-B8908/2014	KX151244
A/Thailand/CU-B8981/2014	KX151245
A/Thailand/CU-B8982/2014	KX151246
A/Thailand/CU-B9024/2014	KX151247
A/Thailand/CU-B9037/2014	KX151248
A/Thailand/CU-B9221/2014	KX151249
A/Thailand/CU-B9225/2014	KX151250
A/Thailand/CU-B9433/2014	KX151251
A/Thailand/CU-H3628/2014	KX151252
A/Thailand/CU-H3632/2014	KX151253
A/Thailand/CU-B10114/2014	KX151254
A/Thailand/CU-C4666/2014	KX151255
A/Thailand/CU-B10032/2014	KX151256

A/Thailand/CU-B10033/2014	KX151257
A/Thailand/CU-B10044/2014	KX151258
A/Thailand/CU-B10126/2014	KX151259
A/Thailand/CU-B10172/2014	KX151260
A/Thailand/CU-B10174/2014	KX151261
A/Thailand/CU-B10180/2014	KX151262
A/Thailand/CU-A1058/2014	KX151263
A/Thailand/CU-B10185/2014	KX151264
A/Thailand/CU-B10207/2014	KX151265
A/Thailand/CU-C4844/2014	KX151266
A/Thailand/CU-A1070/2014	KX151267
A/Thailand/CU-A1105/2014	KX151268
A/Thailand/CU-B10405/2014	KX151269
A/Thailand/CU-B10578/2014	KX151270
A/Thailand/CU-B10652/2014	KX151271
A/Thailand/CU-B10658/2014	KX151272
A/Thailand/CU-B10888/2014	KX151273
A/Thailand/CU-B10909/2014	KX151274
A/Thailand/CU-H3658/2014	KX151275
A/Thailand/CU-C5062/2014	KX151276
A/Thailand/CU-A1205/2014	KX151277
A/Thailand/CU-B11070/2014	KX151278
A/Thailand/CU-C5149/2014	KX151279
A/Thailand/CU-C5169/2014	KX151280
A/Thailand/CU-A1282/2014	KX151281
A/Thailand/CU-B11233/2014	KX151282
A/Thailand/CU-B11291/2014	KX151283
A/Thailand/CU-C5263/2014	KX151284
A/Thailand/CU-A1344/2014	KX151285
A/Thailand/CU-B11363/2014	KX151286
A/Thailand/CU-B11375/2014	KX151287
A/Thailand/CU-B11422/2014	KX151288
A/Thailand/CU-A1434/2015	KX151289
A/Thailand/CU-B11495/2015	KX151290
A/Thailand/CU-B11463/2015	KX151291
A/Thailand/CU-B11556/2015	KX151292
A/Thailand/CU-B11612/2015	KX151293
A/Thailand/CU-B11606/2015	KX151294
A/Thailand/CU-B11669/2015	KX151295
A/Thailand/CU-C5474/2015	KX151296
A/Thailand/CU-B11672/2015	KX151297
A/Thailand/CU-B11699/2015	KX151298
A/Thailand/CU-B11761/2015	KX151299

A/Thailand/CU-C5535/2015	KX151300
A/Thailand/CU-B11797/2015	KX151301
A/Thailand/CU-B11841/2015	KX151302
A/Thailand/CU-B11844/2015	KX151303
A/Thailand/CU-B11877/2015	KX151304
A/Thailand/CU-C5736/2015	KX151305
A/Thailand/CU-B11984/2015	KX151306
A/Thailand/CU-C5866/2015	KX151307
A/Thailand/CU-B12167/2015	KX151308
A/Thailand/CU-B12418/2015	KX151309
A/Thailand/CU-B12591/2015	KX151310
A/Thailand/CU-B12604/2015	KX151311
A/Thailand/CU-B12653/2015	KX151312
A/Thailand/CU-B12974/2015	KX151313
A/Thailand/CU-B13002/2015	KX151314
A/Thailand/CU-B6372/2012	KX151315
A/Thailand/CU-B9280/2014	KX151316
A/Thailand/CU-B9297/2014	KX151317
A/Thailand/CU-H3640/2014	KX151318
A/Thailand/CU-B13202/2015	KX151319
A/Thailand/CU-B13274/2015	KX151320
A/Thailand/CU-B13508/2015	KX151321
A/Thailand/CU-C6326/2015	KX151322
A/Thailand/CU-B14522/2015	KX151323
A/Thailand/CU-B14530/2015	KX151324
A_Bangkok_INS3_681_2012	CY176748
A_Bangkok_INS424_2010	CY071329
A/Bangkok/INS425/2010	CY071337
A/Bangkok/INS426/2010	CY071345
A/Bangkok/INS427/2010	CY071353
A/Bangkok/INS428/2010	CY071361
A/Bangkok/INS477/2010	CY096292
A/Bangkok/INS478/2010	CY096300
A/Bangkok/INS479/2010	CY096308
A/Bangkok/INS480/2010	CY096316
A/Bangkok/INS481/2010	CY096324
A/Bangkok/INS482/2010	CY096332
A/Bangkok/INS483/2010	CY096340
A/Bangkok/INS484/2010	CY098108
A/Bangkok/INS485/2010	CY096348
A/Bangkok/INS486/2010	CY098116
A/Bangkok/INS487/2010	CY098124
A/Bangkok/INS488/2010	CY098132

A/Bangkok/INS489/2010	CY096356
A/Bangkok/INS490/2010	CY098140
A/Bangkok/INS491/2010	CY096364
A/Bangkok/INS492/2010	CY096372
A/Bangkok/INS493/2010	CY096380
A/Bangkok/INS494/2010	CY096388
A/Bangkok/INS495/2010	CY096396
A/Bangkok/INS497/2010	CY096404
A/Bangkok/INS498/2010	CY096412
A/Bangkok/INS499/2010	CY096420
A/Bangkok/INS500/2010	CY096428
A/Bangkok/INS501/2010	CY096436
A/Bangkok/INS502/2010	CY098148
A/Bangkok/INS503/2010	CY096444
A/Bangkok/INS504/2010	CY096452
A/Bangkok/INS505/2010	CY096460
A/Bangkok/INS506/2010	CY096468
A/Bangkok/INS507/2010	CY096476
A/Bangkok/INS508/2010	CY098156
A/Bangkok/INS509/2010	CY098164
A/Bangkok/INS510/2010	CY096484
A/Bangkok/INS511/2010	CY096492
A/Bangkok/INS512/2010	CY096500
A/Bangkok/INS513/2010	CY096508
A/Bangkok/INS514/2010	CY096515
A/Bangkok/INS516/2010	CY098555
A/Bangkok/INS517/2010	CY096523
A/Bangkok/INS518/2010	CY096531
A/Bangkok/INS519/2010	CY096539
A/Bangkok/INS520/2010	CY098565
A/Bangkok/INS580/2010	CY129760
A/Bangkok/INS581/2010	CY129768
A/Bangkok/INS582/2010	CY129776
A/Bangkok/INS583/2010	CY129784
A/Bangkok/INS584/2010	CY129792
A/Bangkok/INS587/2010	CY129816
A/Bangkok/SIMI501/2009	KM013714
A/Bangkok/SIMI502/2010	KM013715
A/Bangkok/SIMI503/2010	KM013716
A/Bangkok/SIMI504/2010	KM013717
A/Bangkok/SIMI505/2010	KM013718
A/Bangkok/SIMI506/2010	KM013719
A/Bangkok/SIMI507/2009	KM013720

A/Bangkok/SIMI508/2010	KM013721
A/Bangkok/SIMI509/2010	KM013722
A/Bangkok/SIMI510/2010	KM013723
A/Bangkok/SIMI511/2010	KM013724
A/Khon Kaen/INS3_640/2010	CY176468
A/Khon Kaen/INS3_647/2010	CY176524
A/Khon Kaen/INS3_649/2012	CY176540
A/Khon Kaen/INS440/2010	CY093058
A/Khon Kaen/INS441/2010	CY096068
A/Khon Kaen/INS443/2010	CY096076
A/Khon Kaen/INS444/2010	CY098204
A/Khon Kaen/INS445/2010	CY096084
A/Khon Kaen/INS446/2010	CY096092
A/Khon Kaen/INS447/2010	CY096100
A/Khon Kaen/INS448/2010	CY093066
A/Khon Kaen/INS449/2010	CY096108
A/Khon Kaen/INS450/2010	CY096116
A/Khon Kaen/INS451/2010	CY096124
A/Khon Kaen/INS452/2010	CY096132
A/Khon Kaen/INS453/2010	CY096140
A/Khon Kaen/INS454/2010	CY096148
A/Khon Kaen/INS455/2010	CY096156
A/Khon Kaen/INS456/2010	CY093074
A/Khon Kaen/INS457/2010	CY096164
A/Khon Kaen/INS458/2010	CY096172
A/Khon Kaen/INS459/2010	CY096180
A/Khon Kaen/INS460/2010	CY096188
A/Khon Kaen/INS461/2010	CY098100
A/Khon Kaen/INS462/2010	CY096196
A/Khon Kaen/INS521/2010	CY096547
A/Khon Kaen/INS522/2010	CY096555
A/Khon Kaen/INS523/2010	CY096563
A/Khon Kaen/INS585/2010	CY129800
A/Khon Kaen/INS586/2010	CY129808
A/Khon Kaen/INS588/2010	CY129824
A/Nonthaburi/102/2009	CY039988
A/Thailand/104/2009	GQ169381
A/Thailand/C602/2010	CY080309
A/Thailand/CU-B2357/2010	CY080301
A/Thailand/CU-B5/2009	GQ866953
A/Thailand/CU-H1786/2010	CY080325
A/Thailand/CU-H1821/2010	CY080341
A/Thailand/CU-H2176/2010	CY088803

A/Thailand/CU-H2283/2010	CY082965
A/Thailand/CU-H2358/2010	CY082966
A/Thailand/CU-H2389/2010	CY082967
A/Thailand/CU-H2417/2010	CY088832
A/Thailand/CU-H2543/2010	CY088840
A/Thailand/CU-FS1/2014	KU051433
A/Thailand/CU-FS2/2014	KU051441
A/Thailand/H1255/2010	CY080317
A/Thailand/H1818/2010	CY080333
A/Thailand/NHRC430218/2014	KJ889263
A/Thailand/SirirajICRC_BKK_1/2010	KF849751
A/Thailand/SirirajICRC_CBI_2/2009	KF849738
A/Thailand/SirirajICRC_CBI_3/2009	KF849739
A/Thailand/SirirajICRC_CBI_4/2009	KF849740
A/Thailand/SirirajICRC_CBI_7/2009	KF849741
A/Thailand/SirirajICRC_CBI_8/2009	KF849742
A/Thailand/SirirajICRC_CBI_9/2009	KF849743
A/Thailand/SirirajICRC_NMA_1/2011	KF849752
A/Thailand/SirirajICRC_NMA_11/2011	KF849761
A/Thailand/SirirajICRC_NMA_12/2011	KF849762
A/Thailand/SirirajICRC_NMA_13/2011	KF849763
A/Thailand/SirirajICRC_NMA_14/2011	KF849764
A/Thailand/SirirajICRC_NMA_15/2011	KF849765
A/Thailand/SirirajICRC_NMA_16/2011	KF849766
A/Thailand/SirirajICRC_NMA_2/2011	KF849753
A/Thailand/SirirajICRC_NMA_3/2011	KF849754
A/Thailand/SirirajICRC_NMA_4/2011	KF849755
A/Thailand/SirirajICRC_NMA_5/2011	KF849756
A/Thailand/SirirajICRC_NMA_6/2011	KF849757
A/Thailand/SirirajICRC_NMA_7/2011	KF849758
A/Thailand/SirirajICRC_NMA_8/2011	KF849759
A/Thailand/SirirajICRC_NMA_9/2011	KF849760
A/Thailand/SirirajICRC_NSN_1/2010	KF849749
A/Thailand/SirirajICRC_NSN_3/2010	KF849750
A/Thailand/SirirajICRC_SEA-001(34)/2010	KF849747
A/Thailand/SirirajICRC_SEA-002(33)/2010	KF849745
A/Thailand/SirirajICRC_SEA-002(34)/2010	KF849744
A/Thailand/SirirajICRC_SEA-003(33)/2010	KF849748
A/Thailand/SirirajICRC_SEA-003(34)/2010	KF849746
A/Thailand/CU-H2548/2010	CY089449
A/Thailand/CU-H2698/2010	CY089457
A/Thailand/CU-H2911/2011	CY089465
A/Thailand/CU-H567/2009	CY074992

A/Thailand/CU-H572/2009	CY075000
A/Thailand/CU-H847/2009	CY075008
A/Thailand/CU-H88/2009	HM446344
A/Thailand/CU-H9/2009	GQ866961
A/Thailand/CU-H910/2009	CY075016
A/Thailand/CU-C1157/2010	CY081157
A/Thailand/CU-C161/2009	CY074976
A/Thailand/CU-H1222/2010	CY074984
A/Thailand/CU-B4148/2010	CY089431
A/Thailand/CU-B4339/2010	CY089439
A/Chanthaburi/38/2015	EPI643493
A/Nonthaburi/53/2015	EPI643329
A/Nonthaburi/52/2015	EPI 643283
A/Nonthaburi/59/2015	EPI 643259
A/Pathumthani/12/2015	EPI636129
A/Chanthaburi/11/2015	EPI636091
A/Nonthaburi/375/2014	EPI565031
A/Nonthaburi/2398/2014	EPI 564983
A/Nonthaburi/72/2013	EPI552434
A/Bangkok/374/2013	EPI552432
A/Song Khla/160/2013	EPI552429
A/Phuket/01/2013	EPI 552427
A/Phuket/316/2014	EPI541523
A/Bangkok/1792/2014	EPI541517
A/SUPHANBURI/234/2014	EPI540902
A/NONTHABURI/248/2014	EPI540897
A/Sukhothai/214/2014	EPI 531877
A/Chanthaburi/190/2014	EPI 531836
A/PRACHUAP KHIRI KHAN/332/2013	EPI 529418
A/Song Khla/139/2014	EPI524286
A/Chiang Rai/157/2013	EPI 466936
A/SONG KHLA/416/2012	EPI 450281
A/Surat Thani/16/2013	EPI 439227
A/Chiang Rai/312/2012	EPI407400
A/Phuket/294/2012	EPI 407397
A/Song Khla/3113/2012	EPI407361
A/Tak/327/2012	EPI406034
A/Chiang Rai/312/2012	EPI 406031
A/Ayutthaya/283/2012	EPI386005
A/Nonthaburi/74/2012	EPI379535
A/SONG KHLA/40/2012	EPI346512
A/Song Khla/270/2011	EPI332584
A/Nonthaburi/78/2011	EPI 331545

A/SONG KHLA/34/2011	EPI 295512
A/Thailand/742/2010	EPI 295494
A/Thailand/594/2010	EPI 276899
A/CHIANG RAI/226/2010	EPI271972
A/SAMUTPRAKAN/102/2010	EPI 231481
A/Surat Thani/563/2009	EPI231343
A/Ayutthaya/568/2009	EPI 221043
A/Thailand/2944/2009	EPI179068

Influenza B

**Virus name**	**Accession number**
B/Victoria/02/1987	CY018759
B/Yamagata/16/1988	CY018767
B/Yamanashi/166/1998	CY019533
B/Lee/40 1940	DQ792899
B/Russia/1969	EF626638
B/Shanghai/361/2002	EF541477
B/Malaysia/2506/2004	CY040451
B/Florida/4/2006 2006	CY033878
B/Brisbane/60/2008	CY115153
B/Wisconsin/01/2010	CY115185
B/Jiangsu/10/2003	CY033846
B/Johannesburg/3964/2012	EPI406273
B/Brisbane/33/2008	CY149983
B/England/393/2008	EPI211560
B/Victoria/304/2006	AGX18593
B/Uruguay/12/2008	EPI172515
B/FujianGulou/1272/2008	EPI366582
B/Singapore/19/2009	EPI193057
B/Argentina/R158/2010	EPI301333
B/Harbin/7/1994	CY040443
B/Sichuan/379/99	AJ784087
B/Bangladesh/3333/2007	AFH58308
B/Stockholm/12/2011	EPI340833
B/Bangkok/141/1994	CY019677
B/Bangkok/153/1990	CY019613
B/Bangkok/163/1990	CY019621
B/Bangkok/34/1999	AY139056
B/Bangkok/54/1999	AY139055

B/Chantaburi/218/2003	CY022223
B/Thailand/SirirajBKK1/2012	KF492944
B/Thailand/SirirajBKK2/2012	KF492945
B/Thailand/SirirajBKK3/2012	KF492946
B/Thailand/SirirajFlu/0006/2012	KF492952
B/Thailand/SirirajFlu/007/2012	KF492953
B/Thailand/SirirajFlu/014/2012	KF492960
B/Thailand/SirirajRMSC1/2010	KF492967
B/Thailand/SirirajEA3604/2008	KF492916
B/Thailand/SirirajSI1335/2010	KF492921
B/Thailand/SirirajSI1648/2010	KF492926
B/Thailand/SirirajSI1659/2010	KF492927
B/Thailand/SirirajSI2117/2010	KF492929
B/Thailand/SirirajSI2210/2010	KF492930
B/Thailand/VIROAF3/2012	KJ577167
B/Thailand/VIROAF3CS/2012	KJ848692
B/Thailand/VIROAF4/2012	KJ577175
B/Bangkok/42/2015	EPI644134
B/Chanthaburi/315/2015	EPI644118
B/Nonthaburi/1/2015	EPI636474
B/Bankgok/22/2015	EPI636306
B/Nonthaburi/373/2014	EPI565142
B/Prachuap/374/2014	EPI562680
B/Nonthaburi/359/2014	EPI562662
B/SongKhla/322/2014	EPI541764
B/Phuket/317/2014	EPI541759
B/ChiangRai/318/2014	EPI541756
B/NongKhai/321/2014	EPI541703
B/CHIANG RAI/273/2014	EPI541360
B/PHUKET/266/2014	EPI541349
B/PRACHUAP/233/2014	EPI541344
B/CHANTHABURI/3046/2013	EPI529350
B/PHUKET/3073/2013	EPI529344
B/TAK/338/2013	EPI529336
B/Tak/145/2014	EPI526088
B/NongKhai/176/2014	EPI526058
B/Sakaeo/73/2014	EPI520499
B/Bangkok/132/2014	EPI520496
B/NongKhai/172/2013	EPI465931
B/ChiangRai/169/2013	EPI465927
B/SongKhla/1598/2013	EPI465914

B/Phuket/151/2013	EPI465899
B/PHUKET/423/2012	EPI450355
B/NONGKAI/419/2012	EPI450349
B/CHIANGRAI/3518/2012	EPI450334
B/ChiangRai/13/2013	EPI433135
B/ChiangRai/15/2013	EPI433126
B/NongKhai/03/2013	EPI431406
B/PRACHUAP/446/2012	EPI417388
B/NONTHANURI/2465/2012	EPI417373
B/NONGKHAI/46/2012	EPI417370
B/NONGKAI/2433/2012	EPI417368
B/CHANTHABURI/2460/2012	EPI417350
B/BANGKOK/29/2012	EPI417329
B/Prachuap/78/2012	EPI408663
B/NongKhai/322/2012	EPI406963
B/Bangkok/282/2012	EPI406956
B/Chanthaburi/318/2012	EPI406950
B/SongKhla/85/2012	EPI387729
B/Phuket/86/2012	EPI387716
B/SONGKHLA/24/2012	EPI379573
B/NONTHABURI/45/2012	EPI379552
B/CHIANGRAI/50/2012	EPI379518
B/PHUKET/53/2012	EPI379515
B/CHIANGRAI/22/2012	EPI379369
B/NONTHABURI/54/2012	EPI379361
B/NONGKHAI/39/2012	EPI379358
B/CHANTHABURI/32/2012	EPI379350
B/Phuket/77/2012	EPI378209
B/NongKhai/67/2012	EPI378206
B/Tak/57/2012	EPI378203
B/ChiangRai/55/2012	EPI378200
B/Bangkok/271/2011	EPI346961
B/NONTHABURI/1766/2011	EPI346107
B/NONGKHAI/112/2011	EPI346104
B/SONGKHLA/43/2011	EPI332672
B/NONGKHAI/40/2011	EPI332669
B/SURATTHANI/29/2011	EPI331536
B/Chanthaburi/74/2011	EPI331117
B/Chanthaburi/73/2011	EPI331114
B/Thailand/820/2010	EPI294862
B/BANGKOK/495/2010	EPI294188

B/ChiangRai/369/2010	EPI278935
B/SONGKHLA/236/2010	EPI272005
B/CHANTHABURI/218/2010	EPI272003
B/SONGKHLA/164/2010	EPI272001
B/NONGKHAI/332/2009	EPI228261
B/CHANTHABURI/329/2009	EPI228259
B/Thailand/1024/2008	EPI176457
B/Thailand/987/2008	EPI176455
B/Thailand/3622/2008	EPI162318
B/Thailand/3365/2008	EPI162316
B/Thailand/393/2008	EPI161761
B/Thailand/374/2008	EPI161759
B/Thailand/373/2008	EPI161757
B/Thailand/94/2007	EPI157243
B/Thailand/89/2007	EPI157241
B/Thailand/869/2007	EPI157239
B/Thailand/810/2007	EPI157237
B/Thailand/81/2007	EPI157235
B/Thailand/762/2007	EPI157232
B/Thailand/264/2007	EPI157228
B/Thailand/1406/2007	EPI157226
B/Phitsanulok/2053/2004	EPI157161
B/Thailand/416/2007	EPI155718
B/Thailand/400/2007	EPI155716
B/Thailand/CU-243/2006	JX513136
B/Thailand/CU-364/2008	JX513144
B/Thailand/CU-A585/2013	KM100243
B/Thailand/CU-A605/2014	KM100244
B/Thailand/CU-A615/2014	KM100247
B/Thailand/CU-A626/2014	KM100248
B/Thailand/CU-A645/2014	KM100251
B/Thailand/CU-B2201/2010	JX512976
B/Thailand/CU-B2271/2010	JX512984
B/Thailand/CU-B2320/2010	JX512992
B/Thailand/CU-B2372/2010	JX513000
B/Thailand/CU-B2390/2010	JX513008
B/Thailand/CU-B2432/2010	JX513016
B/Thailand/CU-B2504/2010	JX513024
B/Thailand/CU-B2660/2010	JX513032
B/Thailand/CU-B3153/2010	JX513040
B/Thailand/CU-B4504/2011	JX513048

B/Thailand/CU-B4585/2011	JX513056
B/Thailand/CU-B5522/2011	JX513064
B/Thailand/CU-B5671/2011	JX513072
B/Thailand/CU-B5734/2011	JX513080
B/Thailand/CU-B5910/2011	JX513088
B/Thailand/CU-B6078/2012	JX513096
B/Thailand/CU-B6096/2012	JX513104
B/Thailand/CU-B6148/2012	KM100232
B/Thailand/CU-B6240/2012	KM100233
B/Thailand/CU-B6257/2012	KM100234
B/Thailand/CU-B6975/2012	KM100237
B/Thailand/CU-B7215/2012	KM100239
B/Thailand/CU-B7337/2012	KM100241
B/Thailand/CU-B8813/2013	KM100242
B/Thailand/CU-B8925/2014	KM100245
B/Thailand/CU-B8999/2014	KM100249
B/Thailand/CU-B9017/2014	KM100250
B/Thailand/CU-B9034/2014	KM100252
B/Thailand/CU-C1262/2010	JX513112
B/Thailand/CU-C1451/2010	JX513120
B/Thailand/CU-C1768/2011	JX513128
B/Thailand/CU-C4555/2014	KM100253
B/Thailand/CU-H1400/2010	JX513152
B/Thailand/CU-H1896/2010	JX513160
B/Thailand/CU-H2132/2010	JX513168
B/Thailand/CU-H2584/2010	JX513176
B/Thailand/CU-H2738/2010	JX513184
B/Thailand/CU-H2933/2011	JX513192
B/Thailand/CU-H3002/2011	JX513200
B/Thailand/CU-H3052/2011	JX513208
B/Thailand/CU-H3313/2012	KM100235
B/Thailand/CU-H3349/2012	KM100236
B/Thailand/CU-H3456/2012	KM100238
B/Thailand/CU-H3496/2012	KM100240
B/Thailand/CU-H3591/2014	KM100246
B/Thailand/CU-H3620/2014	KX151325
B/Thailand/CU-C4610/2014	KX151326
B/Thailand/CU-B9455/2014	KX151327
B/Thailand/CU-B9693/2014	KX151328
B/Thailand/CU-B10007/2014	KX151329
B/Thailand/CU-B10030/2014	KX151330

B/Thailand/CU-A832/2014	KX151331
B/Thailand/CU-B10125/2014	KX151332
B/Thailand/CU-B10127/2014	KX151333
B/Thailand/CU-C4799/2014	KX151334
B/Thailand/CU-B10233/2014	KX151335
B/Thailand/CU-B10235/2014	KX151336
B/Thailand/CU-B10236/2014	KX151337
B/Thailand/CU-B10303/2014	KX151338
B/Thailand/CU-B10340/2014	KX151339
B/Thailand/CU-B10376/2014	KX151340
B/Thailand/CU-B10440/2014	KX151341
B/Thailand/CU-B10508/2014	KX151342
B/Thailand/CU-B10739/2014	KX151343
B/Thailand/CU-B10747/2014	KX151344
B/Thailand/CU-B10835/2014	KX151345
B/Thailand/CU-B11066/2014	KX151346
B/Thailand/CU-B11155/2014	KX151347
B/Thailand/CU-B11168/2014	KX151348
B/Thailand/CU-C5175/2014	KX151349
B/Thailand/CU-B11219/2014	KX151350
B/Thailand/CU-B11259/2014	KX151351
B/Thailand/CU-B11295/2014	KX151352
B/Thailand/CU-B11305/2014	KX151353
B/Thailand/CU-B11371/2014	KX151354
B/Thailand/CU-B11389/2014	KX151355
B/Thailand/CU-B11391/2014	KX151356
B/Thailand/CU-B11425/2014	KX151357
B/Thailand/CU-B11464/2015	KX151358
B/Thailand/CU-B11465/2015	KX151359
B/Thailand/CU-B11472/2015	KX151360
B/Thailand/CU-B11572/2015	KX151361
B/Thailand/CU-B11595/2015	KX151362
B/Thailand/CU-B11603/2015	KX151363
B/Thailand/CU-B11609/2015	KX151364
B/Thailand/CU-B11673/2015	KX151365
B/Thailand/CU-B11681/2015	KX151366
B/Thailand/CU-B11705/2015	KX151367
B/Thailand/CU-B11719/2015	KX151368
B/Thailand/CU-B11774/2015	KX151369
B/Thailand/CU-B11776/2015	KX151370
B/Thailand/CU-B11789/2015	KX151371

B/Thailand/CU-B11823/2015	KX151372
B/Thailand/CU-B11863/2015	KX151373
B/Thailand/CU-B11864/2015	KX151374
B/Thailand/CU-B11901/2015	KX151375
B/Thailand/CU-B11908/2015	KX151376
B/Thailand/CU-B12073/2015	KX151377
B/Thailand/CU-B12092/2015	KX151378
B/Thailand/CU-B12096/2015	KX151379
B/Thailand/CU-B12113/2015	KX151380
B/Thailand/CU-B12741/2015	KX151381
B/Thailand/CU-B12992/2015	KX151382
B/Thailand/CU-B12998/2015	KX151383
B/Thailand/CU-B13258/2015	KX151384
B/Thailand/CU-B13297/2015	KX151385
B/Thailand/CU-B13716/2015	KX151386
B/Thailand/CU-B13874/2015	KX151387
B/Thailand/CU-B13917/2015	KX151388
B/Thailand/CU-B14903/2015	KX151389
B/Thailand/CU-B14300/2015	KX151390
B/Thailand/CU-B14301/2015	KX151391
B/Thailand/CU-B14308/2015	KX151392
B/Thailand/CU-B14349/2015	KX151393
