# Supplementary material for: Evolution of the neuraminidase gene of seasonal influenza A and B viruses in Thailand between 2010 and 2015
Source: PLoS One. 2017 Apr 14;12(4):e0175655. doi: 10.1371/journal.pone.0175655 (PMC5391933; doi:10.1371/journal.pone.0175655)
Supplement: S2 Table — (PDF) [file pone.0175655.s005.pdf]

S2 Table. Clinical data and sequencing results of each patient with oseltamivir resistant strains (275Y) of influenza A H1N1 pdm09

Age	Sex	AA mutation	Ct GAPDH	Ct JOE	Ct FAM	Ratio	Genetic mutant
19	Male	H275Y	29	26	28	4	TAC
32	Female	H275Y	27	9	19	1024	TAC
64	Female	H275Y	29	13	24	2048	TAC
4	Male	H275Y	25	10	17	128	TAC
