# Supplementary material for: Evolution of the neuraminidase gene of seasonal influenza A and B viruses in Thailand between 2010 and 2015
Source: PLoS One. 2017 Apr 14;12(4):e0175655. doi: 10.1371/journal.pone.0175655 (PMC5391933; doi:10.1371/journal.pone.0175655)
Supplement: S3 Table — (PDF) [file pone.0175655.s006.pdf]

S3 Table. Summary of clinical data of 18 patients infected with NAI resistant seasonal influenza viruses infection in the center of excellence in clinical virology, 2009-2015

Viral strain	Age	Gender	AA mutation	Clinical sign [a]	Underlying disease	Duration [b]	LT[c]	complication	Severity [d]
**Influenza A/H1N1 seasonal**									
B589	43	Male	H275Y	Fever, myalgia, rhinorrhea	Grave's disease	1	3	No	Moderate
H17	34	Female	H275Y	Fever, sore throat, productive cough, rhinorrhea, malaise	No	3	2	No	Moderate
H223	34	Female	H275Y	Fever, sore throat, productive cough, rhinorrhea, myalgia	Pregnancy, Allergy; shrimp, Hyperthyroid	No	3	No	Moderate
H565	60	Female	H275Y	Fever, non-productive cough, rhinorrhea, malaise, dizziness, vomiting	Hypertension, Hepatitis B, Asthma, hypercholesterolemia	3	3	No	Moderate
**Influenza A/H1N1 pdm09**									
B6372	19	Male	H275Y	High fever, productive cough, rhinorrhea, malaise, myalgia	Allergy rhinitis	1	3	No	Moderate
B9280	32	Female	H275Y	Fever, sore throat, productive cough, rhinorrhea, myalgia, headache	No	3	2	No	Moderate
B9297	64	Female	H275Y	Fever, productive cough, malaise	No	1	No	No	Mild
H3640	4	Male	H275Y	Fever, non-productive cough, rhinorrhea	Acute lymphoblastic leukemia	3	31	Hypokalemia	Moderate
**Influenza A/H3N2**									
H3435	13	Male	I222V	Fever, sore throat, non-productive cough, rhinorrhea, myalgia, headache	No	3	2	Sinusitis	Moderate
**Influenza B**									
B10340	9	Male	D197N	Fever, myalgia, headache, vomiting, nausea	No	2	No	No	Mild
B7337	55	Female	A395E	Fever, febrile illness	Diabetes Mellitus, Hypertension	4	4	No	Moderate
B5910	50	Male	A395T	Fever, sore throat, non-productive cough	No	No	No	No	Mild
B6148	33	Male	A395T	High fever, sore throat, productive cough, myalgia	No	3	7	No	Moderate
B5522	7	Female	A395D	High fever, non-productive cough, rhinorrhea	No	2	No	No	Mild

B10236	38	Male	A395V	Fever, non-productive cough, myalgia	No	3	3	No	Severe
B2320	33	Male	A395T	Fever, non-productive cough, malaise	Allergy rhinitis	No	5	No	Moderate
H1400	6	Male	A395T	High fever, cough with sputum, rhinorrhea	No	2	7	Sinusitis	Moderate
H3002	7	Male	A395D	High fever, non-productive cough, rhinorrhea, headache, vomiting, depress, loss appetite, diarrhea	No	4	5	No	Moderate

[a] When patients were admitted to hospital

[b] duration from onset of illness to initiation of oseltamivir (days)

[c] Length of hospitalization (days)

[d] OPD cases = mild

Admit = moderate

ICU/O_2 = Severe
