# Supplementary material for: Evolution of the neuraminidase gene of seasonal influenza A and B viruses in Thailand between 2010 and 2015
Source: PLoS One. 2017 Apr 14;12(4):e0175655. doi: 10.1371/journal.pone.0175655 (PMC5391933; doi:10.1371/journal.pone.0175655)
Supplement: S4 Table — (PDF) [file pone.0175655.s007.pdf]

S4A Table. Positively selected sites on the neuraminidase of influenza A/H1N1 pdm09 among strains isolated in Thailand, 2009-2015.

**Codon**	**SLAC d*N*-d*S***	**SLAC *p*-value**	**FEL d*N*-d*S***	**FEL *p*-value**	**MEME ω⁺**	**MEME *p*-value**
13	6.329	0.133	3.959	**0.039**	>100	**0.056**
34	6.201	0.148	4.239	**0.057**	>100	**0.078**
48	6.344	0.132	4.27	**0.074**	>100	**0.098**
188	4.888	0.23	3.542	**0.059**	>100	**0.079**
270	5.86	0.2	3.981	**0.093**	>100	0.118
366	2.727	0.479	1.531	0.62	>100	**0.009**
451	4.691	0.271	2.776	**0.098**	>100	0.124
463	8.239	**0.033**	5.886	**0.013**	>100	**0.000**

S4B Table. Positively selected sites on the neuraminidase of seasonal influenza A/H1N1 among strains isolated in Thailand, 2009-2015.

**Codon**	**SLAC d*N*-d*S***	**SLAC *p*-value**	**FEL d*N*-d*S***	**FEL *p*-value**	**MEME ω⁺**	**MEME *p*-value**
7	-7.864	0.998	-27.072	0.084	>100	**0.012**
77	3.657	**0.088**	11.702	**0.079**	>100	**0.036**
222	8.798	**0.006**	33.293	**0.006**	>100	**0.01**
249	2.451	0.199	7.726	**0.074**	>100	**0.097**
266	1.93	0.274	9.57	0.121	>100	**0.045**
344	2.767	0.193	9.633	**0.055**	>100	**0.075**
452	5.606	**0.033**	19.616	**0.018**	>100	**0.024**

S4C Table. Positively selected sites on the neuraminidase of influenza A/H3N2 among strains isolated in Thailand

Codon	SLAC d*N*-d*S*	SLAC *p*-value	FEL d*N*-d*S*	FEL *p*-value	MEME ω^+	MEME *p*-value
4	-5.582	0.988	-3.225	**0.047**	>100	**0**
43	3.88	0.179	2.196	**0.082**	41.261	**0.02**
93	4.092	**0.078**	1.601	**0.038**	>100	**0.055**
141	3.263	0.166	1.649	**0.038**	>100	**0.054**
150	2.369	0.214	1.157	**0.096**	>100	0.12
181	-0.529	0.87	-0.337	0.672	>100	**0**
215	3.586	**0.094**	1.72	**0.039**	>100	**0.056**
267	1.097	0.492	0.221	0.87	66.257	**0.023**
271	2.72	0.223	1.264	0.106	>100	**0.009**
401	3.602	**0.091**	1.53	**0.049**	>100	**0.068**
402	2.175	0.302	1.059	0.137	>100	**0.088**
464	2.972	0.143	1.562	**0.032**	>100	**0.001**
468	3.623	**0.088**	1.837	**0.032**	>100	**0.046**

S4D Table. Positively selected sites on the neuraminidase of influenza B among strains isolated in Thailand

**Codon**	**SLAC d*N*-d*S***	**SLAC *p*-value**	**FEL d*N*-d*S***	**FEL *p*-value**	**IFEL d*N*-d*S***	**IFEL *p*-value**	**MEME ω^{+}**	**MEME *p*-value**
15	-	-	-	-	0.313	0.024	>100	0.005
27	-	-	-	-	0.321	0.018	>100	0.04
41	-	-	-	-	0.151	0.073	>100	0.007
51	-	-	-	-	0.244	0.067	>100	0.077
68	-	-	0.162	0.096	0.228	0.078	-	-
73	0.447	0.036	0.483	0.058	-	-	14.964	0.033
106	-	-	0.365	0.055	0.387	0.090	5.167	0.075
107	-	-	-	-	0.280	0.064	>100	0.085
219	-	-	-	-	0.144	0.066	>100	0.011
220	-	-	0.113	0.051	0.216	0.027	>100	0.092
248	-	-	0.153	0.056	0.220	0.047	-	-
358	-	-	0.363	0.04	0.446	0.035	>100	0.052
395	0.633	0.002	0.615	0.002	0.646	0.008	>100	0.004
404	0.284	0.092	0.255	0.085	0.690	0.009	>100	0.035
465	-	-	0.237	0.093	-	-	>100	0.099

d*N*/d*S* or ω is the ratio of synonymous to non-synonymous substitutions.

p-value from the SLAC, FEL, and MEME results (for positive selective pressure).

Statistically significant values are reported in bold.
